# SHCBP1 is a novel regulator of PLK1 phosphorylation and promotes prostate cancer bone metastasis

**DOI:** 10.1002/mco2.70082

**Published:** 2025-02-13

**Authors:** Chen Tang, Shengmeng Peng, Yongming Chen, Bisheng Cheng, Shurui Li, Jie Zhou, Yongxin Wu, Lingfeng Li, Haitao Zhong, Zhenghui Guo, Yiming Lai, Hai Huang

**Affiliations:** ^1^ Department of Urology Sun Yat‐sen University, Sun Yat‐sen Memorial Hospital Guangzhou P.R. China; ^2^ Guangdong Provincial Key Laboratory of Malignant Tumor Epigenetics and Gene Regulation Sun Yat‐sen Memorial Hospital, Sun Yat‐sen University Guangzhou P.R. China; ^3^ Department of Urology Shenzhen Nanshan People's Hospital Shenzhen P.R. China; ^4^ Guangdong Provincial Clinical Research Center for Urological Diseases Guangzhou P.R. China; ^5^ Beijing Hospital, National Center of Gerontology Institute of Geriatric Medicine Chinese Academy of Medical Sciences & Peking Union Medical College Beijing P.R. China; ^6^ Department of Urology the Fifth Affiliated Hospital of Xinjiang Medical University Urumqi Xinjiang P.R. China

**Keywords:** bone metastasis, PLK1, prostate cancer, SHCBP1

## Abstract

Prostate cancer is a common male genitourinary malignancy with bone metastasis posing challenges for prognosis and treatment. This study aimed to investigate the role of SHC protein SH2 structural domain binding protein 1 (SHCBP1) in prostate cancer bone metastasis. Whole transcriptome sequencing of prostate cancer samples was conducted to identify oncogene expression, specifically focusing on SHCBP1. In vivo and in vitro models were used to study SHCBP1's impact on bone metastasis. Through co‐immunoprecipitation, mass spectrometry, and Western blot assays, the interaction between SHCBP1 and cell cycle‐related proteins was elucidated, along with analysis of downstream protein partners. SHCBP1 was found to enhance prostate cancer cell development, metastasis, and mitosis, with the SHCBP1—polo‐like kinase 1 (PLK1)—CDC25C axis playing a key role in promoting tumorigenesis. Therapeutic inhibition of SHCBP1 increased docetaxel sensitivity. Clinical data showed elevated SHCBP1 expression in advanced prostate cancer stages. These findings offer insights into potential therapeutic strategies for prostate cancer bone metastasis and highlight the significance of the SHCBP1‐PLK1‐CDC25C axis in docetaxel sensitivity.

## Graphical abstract

Model for the interaction of SHCBP1 and PLK1. Schematics summarizing the interaction between SHCBP1 and PLK1, which promotes the Aurora A‐PLK1 interaction, leading to altered phosphorylation of PLK1 at T210, thus promoting cell mitosis and contributing to tumor development via the SHCBP1‐PLK1‐CDC25C axis. It also highlights the involvement of SHCBP1 in epithelial–mesenchymal transition (EMT), facilitating cancer cell metastasis, and docetaxel chemoresistant.

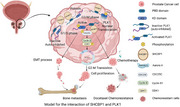

## 1 INTRODUCTION

The most frequent type of male genitourinary cancer is prostate cancer (PCa), which is the fifth most deadly disease overall. For localized PCa, the 5‐year survival rate is 100%, but for advanced PCa, it is 30.6%.^1^ Ninety percent of PCa metastases occur in axial bones, such as those of the spine.^2^ Pathologic fractures and spinal cord compression are important skeletal events associated with bone metastases that can reduce patients’ quality of life, physical function, and mobility. PCa bone metastasis is driven by increased cell proliferation and metastatic ability. Current therapies are ineffective after PCa bone metastasis, and the regulatory mechanisms are uncertain. Recent studies have shown that tumor cells that form bone metastases do not all originate from the same primary tumor site. The bone environment can alter PCa cell epigenetics, favoring metastasis and secondary metastasis to other organs.^3^ In the rat spontaneous tumorigenesis model, bone metastasis usually comes first, followed by organ metastases, and some of the tumor cells of organ metastases are from bone metastases.^4^ Consequently, the occurrence of bone metastasis can exacerbate the condition of PCa; therefore, identifying effective targets for PCa bone metastasis is of great value for both prevention and therapy.

Normal cell cycles include five phases: dormancy (G0), pre‐DNA replication (G1), DNA replication (S), post‐DNA replication (G2), and mitosis (M). Abnormal cell cycle progression is an important feature of the development of various tumors, and tumor cells in metastatic sites have a stronger ability to rapidly proliferate.^5^ Multiple studies have revealed that aberrant cell mitotic cycle including both G1/S and G2/M transition has been linked to tumor metastasis, and cell cycle proteins regulate both the cell cycle and tumor cell metastasis. By regulating the G1/S phase, cyclin‐dependent kinase 4 (CDK4) and CDK6 may participate in breast cancer cell metastasis,^6^ high Cyclin D1/CDK4 complex levels increase glioblastoma cell metastasis,^7^ and RUNX2 promotes PCa cell bone metastases and tissue invasion of C4‐2B and LNCap PCa cells.^8^ The cytokine inhibitory proteins p16, p21, and p27 also affect tumor cell metastasis.^9^, ^10^, ^11^, ^12^ In addition, the WD repeat‐containing protein 4 (WDR4) promotes G2/M phase transition and hepatocellular carcinoma metastasis by binding eukaryotic initiation translation factor 2A (EIF2A) to Cyclin B1 (CCNB1) mRNA to increase CCNB1 translation.^13^ In primary tumors and lymph node metastases, immune cells and tumor stromal cells express similar genes, but tumor cells differ in terms of the expression of genes correlated with the cell cycle, hypoxic stress, partial epithelial–mesenchymal transition (EMT), and epithelial differentiation.^14^ Multicenter genomic and transcriptomic sequencing of 224 primary and 95 metastatic foci from 289 pancreatic ductal carcinoma patients demonstrated that sequential tumor suppressor inactivation accelerates cell cycle transition and is probably mediated by cell cycle regulatory gene variations.^5^ By swath proteomics sequencing of PCa primary and bone metastatic tissues, Iglesias‐Gato et al.^15^ found that a subset of BM2 PCa cells presented upregulation of genes associated with the cell cycle, DNA damage repair, and cell proliferation. In addition, the reactivation of tumor cell dormancy can make cancers more aggressive and drug‐resistant and accelerate primary tumors and metastases.^16^

Phosphorylated polo‐like kinase 1 (PLK1) is critical for regulating the G2/M phase.^17^ G2/M checkpoint‐related genes are enriched in breast cancer bone metastases, and PLK1 expression is upregulated and promotes tumor cell proliferation.^18^ The transition from primary prostate cancer to metastatic prostate cancer involves upregulation of DNA replication, mitosis, and PLK1 phosphorylation‐mediated events.^19^ PLK1 is minimally expressed in normal prostatic glands but is upregulated in prostate, breast, colorectal, pancreatic, and malignant melanoma. PLK1 is highly expressed in castration‐resistant PCa (CRPC) cell lines. Reagan et al.^20^ found high PLK1 expression in CRPC cells such as prostate cancer cell line 3 (PC3), Duke University 145 (DU145), and lymph node carcinoma of the prostate (LnCAP), but not in prostate epithelial cells (PrEC) normal prostate epithelial cells, and PLK1 knockdown in CRPC cells caused cell cycle arrest but not in prostate epithelial cells. In CRPC cells, CHK1 and PLK1 inhibitors increased cell apoptosis in docetaxel‐resistant cells.^21^ Elodie Montaudon et al.^18^ found that G2/M checkpoint‐related genes were enriched in bone metastases, and PLK1 expression was elevated in paired breast cancer xenograft models. PLK1 downregulation may contribute to PCa bone metastatic dormancy.^22^ BVSK Chakravarthi et al.^23^ discovered that the SUB1 protein is capable of binding to the PLK1 promoter region to increase PCa cell development and metastasis in vitro, and inhibition of SUB1 interferes development and metastasis of PCa in vivo. However, it is not clear how PLK1 phosphorylation is regulated in prostate cancer bone metastasis.

The cell surface receptor protein Src homolog (SHC) activates many growth factor receptors (FGFR, EGFR, IGFR, etc.) via multiple signaling pathways, and the SHCBP1 activates the downstream pathway. Eri Asano et al.^24^ revealed that Aurora B phosphorylates Ser634 of SHCBP1 during mitosis in ovarian cancer and promotes tumor spread by inhibiting MgcRacGAP‐mediated Rac1 inactivation. SHCBP1 promotes gastric cancer multiplication and metastasis and is a viable therapeutic target.^25^ SHCBP1 induces the EGFR signaling pathway to promote β‐catenin nuclear translocation and NSCLC development.^26^ SHCBP1's significance in PCa bone metastases is unclear. Here, we discovered that SHCBP1 regulates PLK1 phosphorylation to regulate PCa cell proliferation, metastasis, and G2/M phase transition. Targeted inhibition of SHCBP1‐PLK1 may inhibit the proliferation and metastasis of prostate cancer cells and enhance the chemotherapeutic efficacy of docetaxel in PCa cells. SHCBP1 has prospective clinical applications in the therapy treatment of PCa.

## 2 RESULTS

### 2.1 Genes involved in the G2/M phase of the cell cycle were upregulated during the bone metastasis of PCa

**FIGURE 1:**
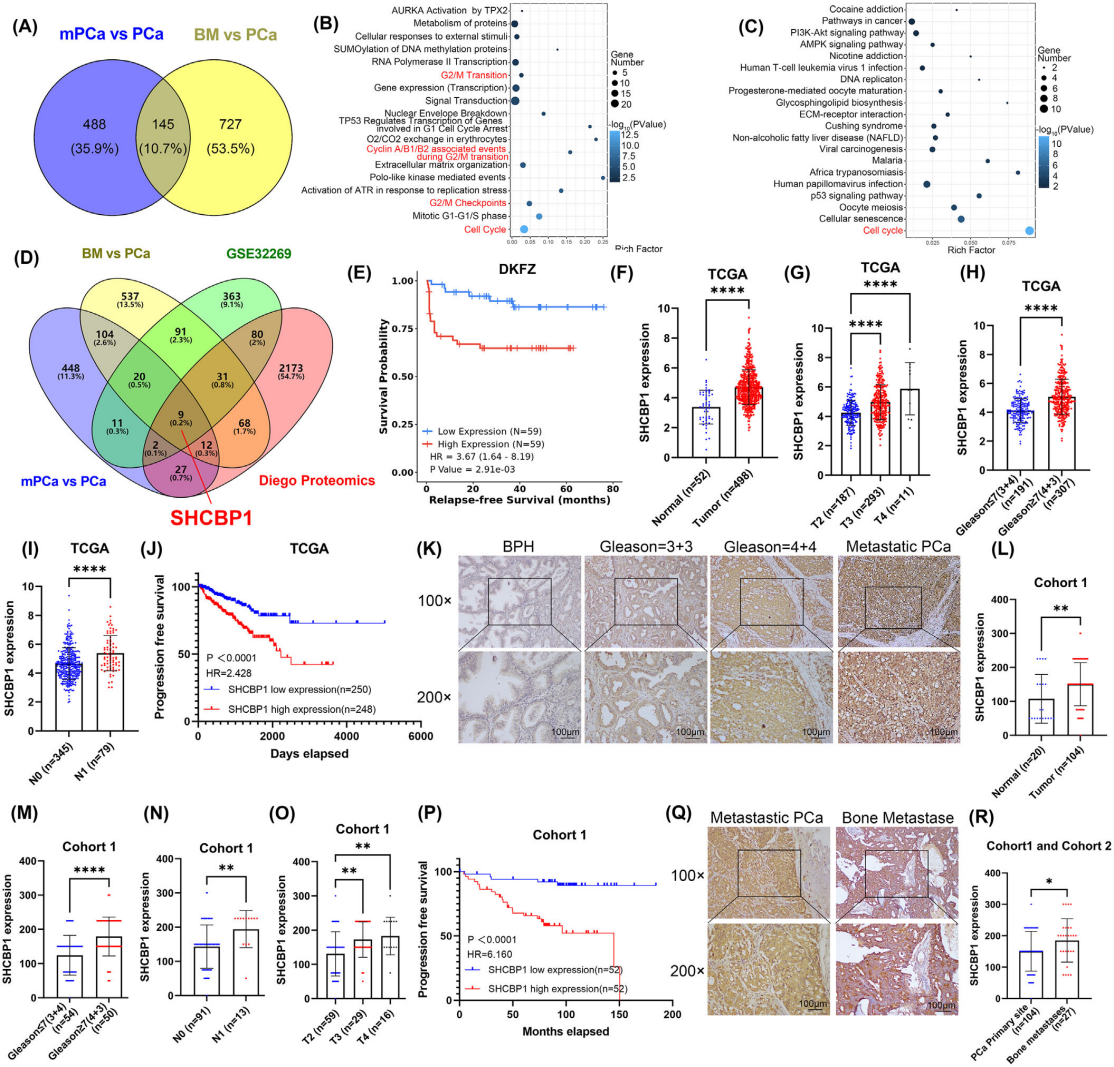
A high level of SHCBP1 expression in metastatic PCa tissue is correlated with a poor prognosis. (A) Venn diagram of upregulated DEGs in the RNA sequencing analysis of the SYSU cohort. (B) Bubble map KEGG pathway enrichment analysis of 145 differentially upregulated genes in (A). (C) Bubble map of Reactome pathway enrichment analysis of 145 differentially upregulated genes in (A). (D) The intersection Venn diagram of SYSU‐RNA sequencing, GSE32269, and Diego proteomics. (E) Progression‐free survival (PFS) of patients in the Deutsches Krebsforschungszentrum (DKFZ) database sorted based on SHCBP1 expression. (F) The differential expression of SHCBP1 in normal prostate tissues and PCa tissues in the TCGA‐PRAD database. (G) The differential expression of SHCBP1 with different T stages. (H) The differential expression of SHCBP1 between PCa tissues with different Gleason scores. (I) The differential expression of SHCBP1 in lymph node‐negative (N0) and lymph node‐positive (N1) PCa. (J) Kaplan‒Meier curves for PFI for patients with various SHCBP1 levels in the TCGA database. (K) Typical images of SHCBP1 expression in BPH, primary PCa with low and high Gleason scores, and metastatic PCa. Scale bars measure 100 µm. (L) The differential expression of SHCBP1 in BPH tissues and PCa tissues in Cohort 1. (M) The differential expression of SHCBP1 between PCa tissues with different Gleason scores in Cohort 1. (N) The differential expression of SHCBP1 in lymph node‐negative (N0) and ‐positive (N1) PCa in Cohort 1. (O) The differential expression of SHCBP1 with different T stages in Cohort 1. (P) Kaplan‒Meier curves for progression‐free survival for patients with various SHCBP1 levels in Cohort 1. (Q) Typical images of SHCBP1 expression in metastatic PCa and bone metastases. Scale bars measure 100 µm. (R) The differential expression of SHCBP1 between primary PCa and bone metastases in cohort 1 and cohort 2. Values are expressed as mean ± SD, *p < 0.05, **p < 0.01, ***p < 0.001, ****p < 0.0001.

We previously conducted whole‐transcriptome sequencing of 13 PCa tissues (7 primary PCa, 6 bone metastatic PCa in situ (bmPCa), and 6 bone metastases of PCa) to identify oncogenes in the process of bone metastasis (BM).^27^ Figure 1A shows that 866 elevated genes were discovered in the BM versus PCa group, and 632 were found in the bmPCa versus PCa group; these gene sets shared 145 common genes. Further enrichment analysis of KEGG and Reactome pathways was performed to determine the biological mechanism underlying these 145 upregulated genes in PCa. These genes were abundant in cell cycle‐related alterations, especially in the G2/M phase (Figure 1B,C; Figure S1A,B).

### 2.2 PCa with high expression of SHCBP1 is associated with advanced clinicopathological features and poor prognosis

To screen for upstream regulators in PCa bone metastasis, we performed an intersection analysis of our sequencing results with the GEO database (GSE32269) and proteomic sequencing (Diego proteomics) results to identify key regulatory genes. PCa bone metastases presented higher HBB, FN1, CDK1, HBA1, MCM4, SHCBP1, FABP4, TFRC, and TK1 expression than primary PCa (Figure 1D). Only CDK1, SHCBP1, and TK1 showed a positive association with a poor prognosis in Kaplan‒Meier analysis in the Deutsches Krebsforschungszentrum database (Figure 1E; Figure S2). In‐depth studies have shown that CDK1 regulates tumor cell cycle control, and TK1, a rate‐limiting enzyme for DNA repair synthesis, is a known S‐phase‐dependent enzyme of the cell cycle and has been widely used as a serum marker for tumor cell proliferation dynamics. Therefore, we investigated the biochemical and clinical implications of SHCBP1 in PCa.

First, we examined the expression and clinicopathologic features of SHCBP1 in the TCGA‐PRAD database to determine its clinical relevance in PCa (Table S1). Figure 1F shows that PCa expressed significantly higher levels of SHCBP1 than normal or benign prostate tissues. High SHCBP1 expression was associated with worse clinicopathological features, such as higher T stage and advanced Gleason score (Figure 1G,H). Patients with lymph node positivity had increased SHCBP1 expression (Figure 1I). PCa patients with high SHCBP1 expression had shorter progression‐free intervals according to Kaplan‒Meier analyses (Figure 1J). High SHCBP1 transcript levels independently increased PCa risk in univariate and multivariate Cox regression models (Table S2).

SHCBP1 expression in paraffin‐embedded human tissue was examined using immunohistochemistry. We collected samples from 20 benign prostatic hyperplasia (BPH) patients, 104 PCa patients (Cohort 1, in situ), and 27 PCa bone metastasis patients (Cohort 2, BM). PCa had a higher SHCBP1 immunoreactivity score (IRS) than BPH (Figure 1L). SHCBP1 expression was significantly associated not only with higher T stage and advanced Gleason score but also with increased metastatic potential and poor prognosis (Figure 1K–P). SHCBP1 protein levels independently increased PCa risk in univariate and multivariate Cox regression models (Table S3). Bone metastases exhibited a considerably higher SHCBP1 IRS score than PCa in situ tissues (Figure 1Q,R). In conclusion, SHCBP1 may be a clinical biomarker for PCa since it is associated with unfavorable clinicopathologic features and a poor prognosis.

### 2.3 SHCBP1 promotes the proliferation and G2/M phase progression of PCa cells in vitro

**FIGURE 2:**
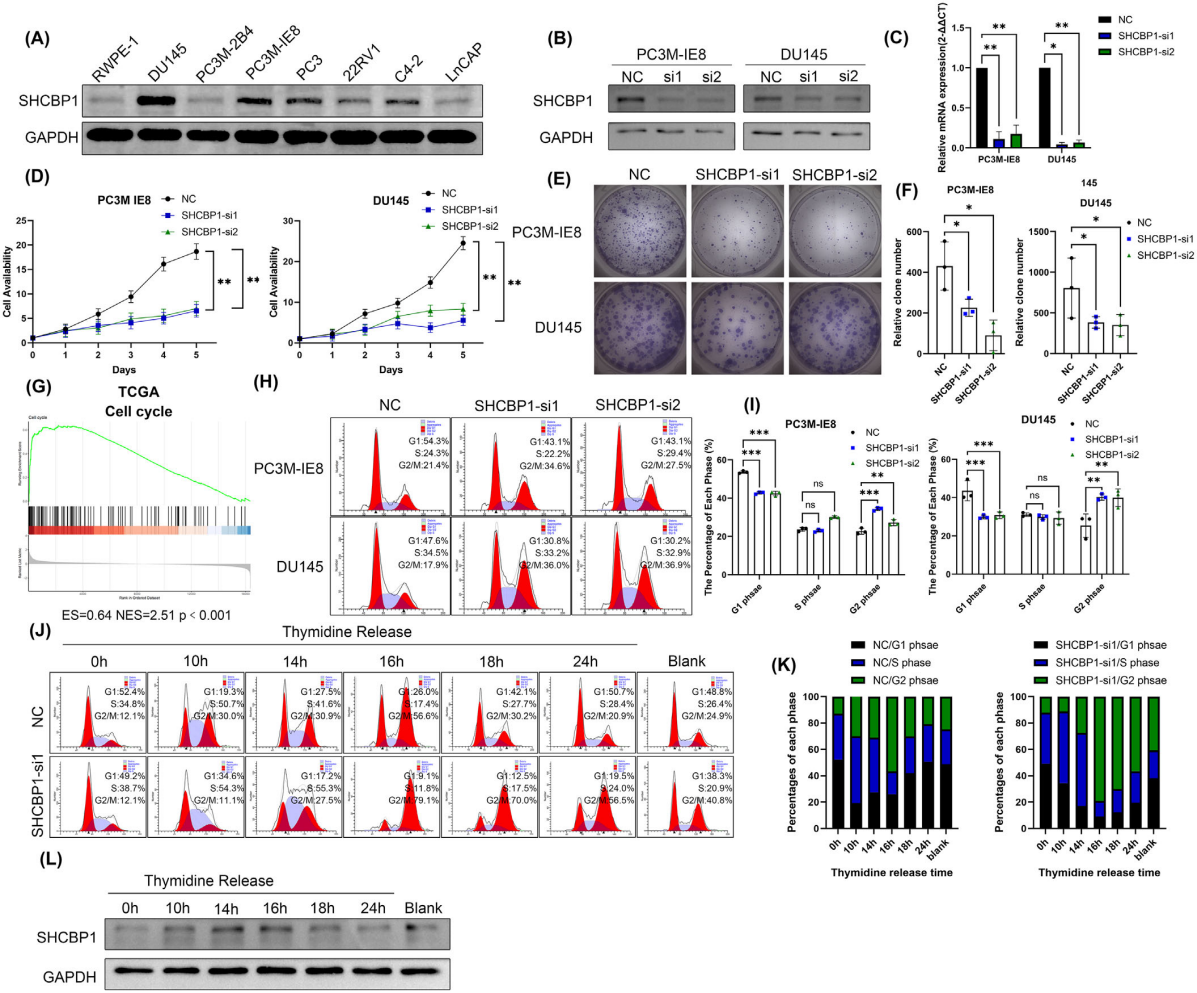
SHCBP1 promotes the proliferation and G2/M phase progression of PCa cells. (A) SHCBP1 expression in PCa and normal prostate epithelial cells. (B) Protein levels of SHCBP1 knockdown cells. (C) mRNA levels of SHCBP1 knockdown cells. (D) Cell viability of SHCBP1 knockdown cells. (E) Colony formation assays of SHCBP1 knockdown cells. (F) Histograms of the colony formation assay. (G) GSEA was used to analyze the potential functions of SHCBP1 in TCGA‐PRAD datasets. (H) Representative images of cell cycle analysis of SHCBP1 knockdown cells. (I) Histograms of cell cycle analysis of SHCBP1 knockdown cells. (J) Cell cycle analysis of SHCBP1 knockdown cells released after synchronization by thymidine (2 nM). (K) Histograms of cell cycle analysis in each phase. (L) SHCBP1 expression followed by thymidine synchronization and release at the indicated time. Values are expressed as mean ± SD, *n* = 3/group in (C–F, I). *p < 0.05, **p < 0.01, ***p < 0.001.

**FIGURE 3:**
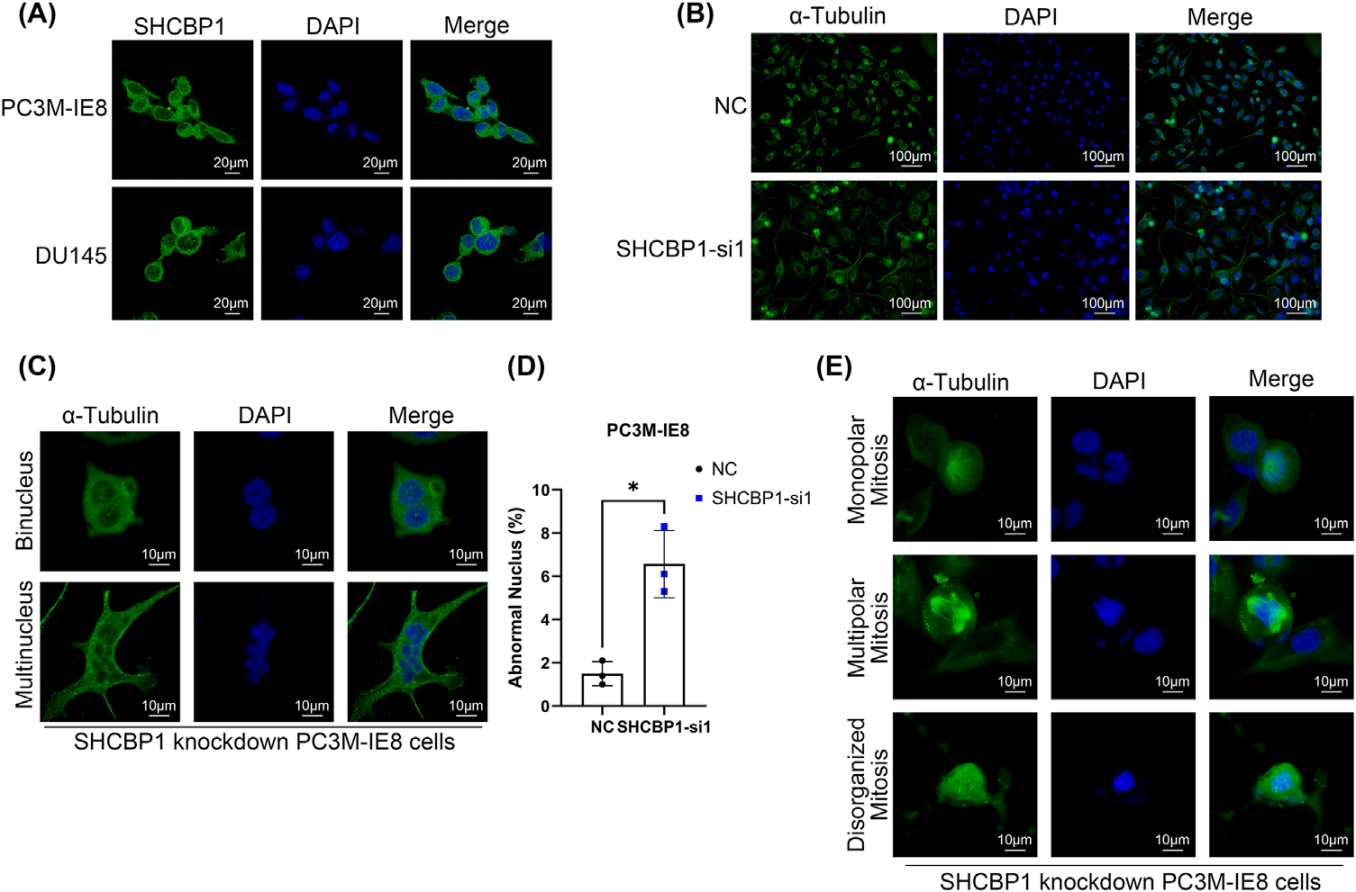
Knockdown of SHCBP1 leads to disruption of PCa cell mitosis. (A) Immunofluorescence of SHCBP1 in PC3M‐IE8 and DU145 cells indicating SHCBP1. (B) Mitotic arrest and abnormal nuclear division in PC3M‐IE8 cells after SHCBP1 knockdown. (C) Representative PC3M‐IE8 abnormally polyploid cells after SHCBP1 knockdown. (D) Statistical plot of percentage of abnormal nuclei after knockdown SHCBP1, *n* = 3/group. (E) Representative images of abnormal microtubule polarization after SHCBP1 knockdown.

Our previous studies suggest that an abnormal cell cycle progression rate and uncontrolled cell proliferation are essential in PCa bone metastasis. PC3M‐IE8 and DU145 PCa cells expressed the highest level of SHCBP1 (Figure 2A). We speculate that SHCBP1 may promote PCa cell growth and metastasis. To test this theory, we designed two small interfering RNAs (siRNAs) targeting different protein coding regions (coding DNA sequence [CDS] regions) of SHCBP1 and subsequently knocked down SHCBP1 in PC3M‐IE8 and DU145 cells. Western blotting and RT‐qPCR showed that these two siRNAs effectively reduced SHCBP1 protein and mRNA levels in PC3M‐IE8 and DU145 cells (Figure 2B,C). SHCBP1 knockdown reduced PC3M‐IE8 and DU145 PCa cell proliferation (Figure 2D) and colony formation (Figure 2E,F). SHCBP1 was also associated with PCa and the cell cycle in the TCGA database (Figure 2G). Cell cycle progression is an important factor influencing the rate of cell proliferation. We observed G2/M phase arrest in SHCBP1 knockdown cells (Figure 2H,I). In SHCBP1 knockdown cells, the rate of transition from G1 to G2 was unaffected, but the transition from G2 to the next cell cycle was significantly slowed (Figure 2J,K). In addition, SHCBP1 expression was suppressed during G1, increased during S, peaked during G2/M, and declined rapidly thereafter (Figure 2L). Immunofluorescence was used to localize SHCBP1 in PC3M‐IE8 and DU145 PCa cells. During interphase of mitosis, SHCBP1 was mostly in the cytoplasm but was also present in the nucleus (Figure 3A). We downregulated the expression of SHCBP1 by means of siRNA interference, in the SHCBP1 knockdown group, significantly more cells within abnormal mitosis were observed (Figure 3B), corroborating the flow cytometry results. Polyploid cells, such as binuclear and multinuclear cells and cells with abnormal nuclear morphology, increased significantly (Figure 3C,D). We also observed more abnormal chromosome division, microtubule assembly, and polarity disorders in cells in mitosis and an increased proportion of cells with unipolar, multipolar, or disorganized division (Figure 3D).

### 2.4 SHCBP1 promotes PCa bone metastasis in vivo

**FIGURE 4:**
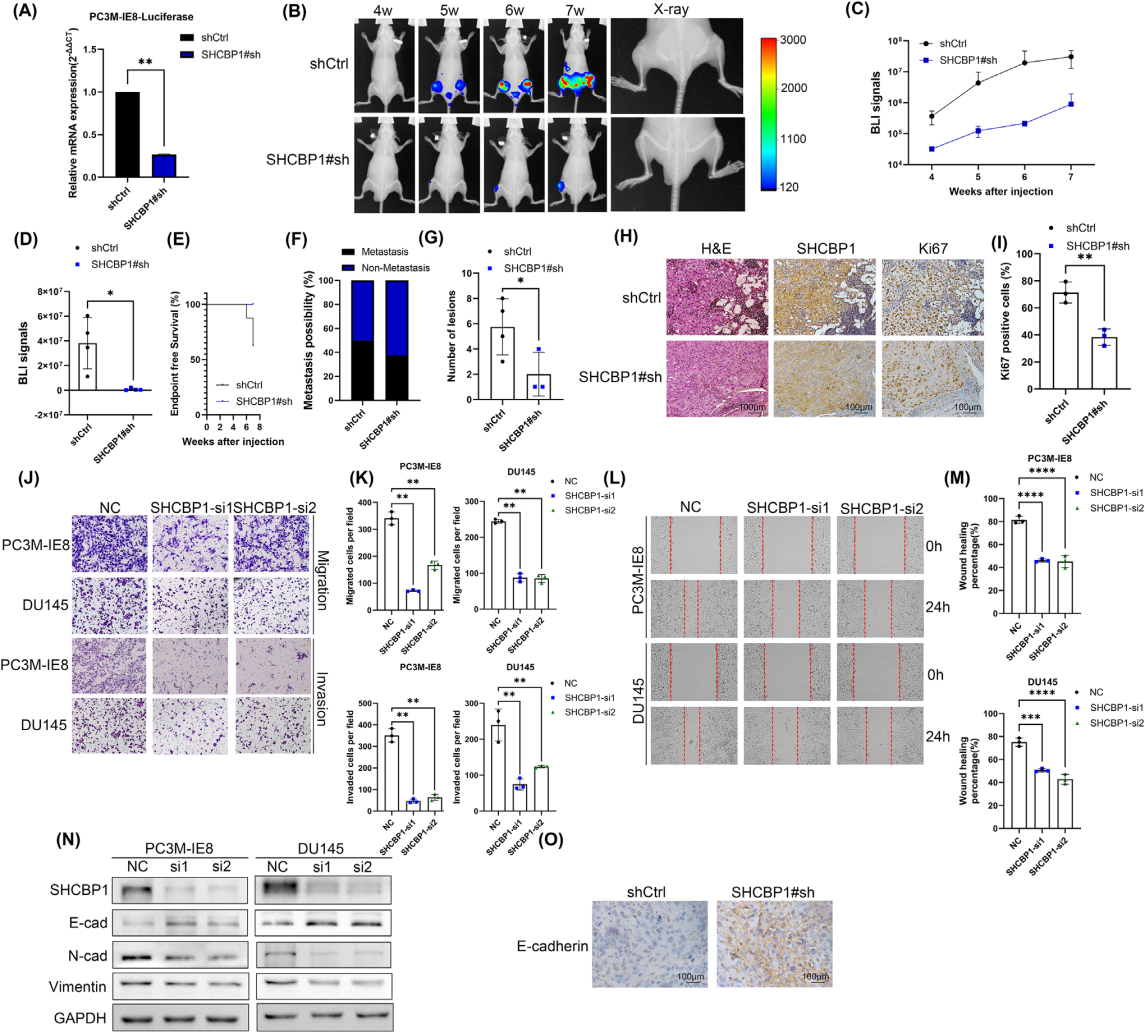
SHCBP1 promotes PCa cell metastasis both in vivo and in vitro. (A) mRNA levels of SHCBP1 in stable knockdown SHCBP1#sh PC3M‐IE8‐luciferase cells. (B) Bone metastasis bioluminescence (BLI) and X‐ray images in nude mice following caudal artery injection with PC‐3 M‐IE8‐luciferase SHCBP1#sh or negative control cell lines, *n* = 8 mice/group, median BLI signal from (C). (C) BLI signals of nude mice at the indicated time. (D) Bone metastasis BLI signals in each group. (E) Kaplan‒Meier curves of the endpoint in each group. (F) Percentage of groups with bone metastases, 4 of 8 in shCtrl group and 3 of 8 in SHCBP1#sh group generated bone metastases. (G) Histograms depicting the total number of bone metastases found in each group, *n* = 4 in the shCtrl group and *n* = 3 in the SHCBP1#sh group. (H) Representative HE and immunohistochemical images of SHCBP1 and Ki67 in bone metastases. (I) Histograms of the Ki67‐positive rate in each group. (J, K) SHCBP1 knockdown cell migration and invasion assay images (J) and histograms (K). (L, M) SHCBP1 knockdown cell wound healing assay images (L) and histograms (M), *n* = 3/group (N) EMT‐related proteins in SHCBP1 knockdown cells. (O) Representative immunohistochemical images of E‐cadherin in bone metastases. *p < 0.05, **p < 0.01.

To determine the impact of SHCBP1 on PCa bone metastases in vivo, we further constructed PC3M‐IE8‐luciferase cells with stable silencing of the expression of SHCBP1 mediated by lentivirus (SHCBP1#sh). RT‐qPCR confirmed that SHCBP1 was knocked down in PC3M‐IE8‐luciferase SHCBP1#sh cells (Figure 4A). We injected 2 × 10^6^ PC3M‐IE8‐luciferase cells into the tail arteries of nude mice to generate bone metastasis xenografts based on previous studies.^28^ Four mice in the control group and three mice in the SHCBP1 knockdown group generated bone metastases. SHCBP1 knockdown not only reduced bioluminescence imaging (BLI) signals (Figure 4B–D; Figure S5A) but also reduced the bone metastasis formation rate (Figure 4F), but the end‐point survival of each group was equivalent (Figure 4E). In addition, X‐ray and H&E staining revealed that SHCBP1 knockdown reduced bone lesions (Figure 4G,H). Immunohistochemistry (staining showed that SHCBP1 expression and the Ki67‐positive rate were lower in the SHCBP1 knockdown group (Figure 4H; Figure S5B).

### 2.5 SHCBP1 promotes the metastatic behavior of PCa cells by regulating the EMT process

The SHCBP1 knockdown group had considerably decreased metastase volume, bone metastasis percentage, and bone tissue destruction, indicating that SHCBP1 may regulate the cell cycle and proliferation as well as cell migration and invasion. We performed transwell assays, and results showed that SHCBP1 knockdown dramatically reduced PC3M‐IE8 and DU145 cell migration and invasion (Figure 4J,K), and the wound healing assay also demonstrated the impairment of wound healing ability by SHCBP1 knockdown (Figure 4L,M). Tumor cells that undergo EMT can become more metastatic. During the process of EMT, tumor cells acquire higher migration and invasion potential and lose mesenchymal features. Western blot assays were used to evaluate epithelial and mesenchymal markers. SHCBP1 knockdown boosted E‐cadherin and reduced N‐ and vimentin (Figure 4N). Furthermore, in our bone metastasis xenograft, the expression of E‐cadherin in dissected bone metastases was restored in the SHCBP1 knockdown group (Figure 4O; Figure S5C). Based on the foregoing findings, SHCBP1 likely regulates PCa cell EMT to promote metastasis.

### 2.6 SHCBP1 regulates the PCa cell cycle and proliferation through interaction with wild‐type PLK1 but not PLK1 mutant T210D

**FIGURE 5:**
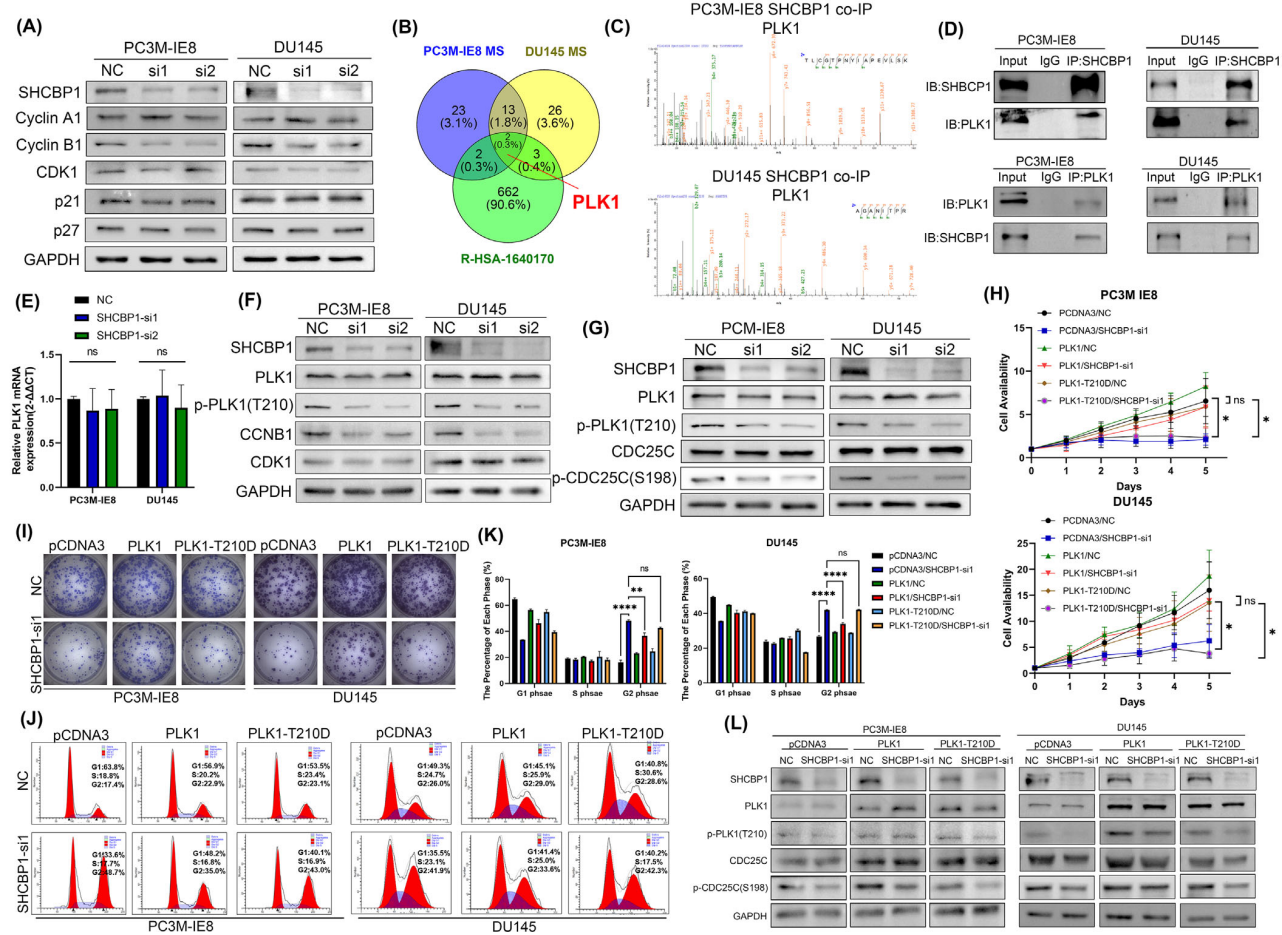
SHCBP1 regulates the PCa cell cycle by regulating PLK1 phosphorylation. (A) Western blot analysis of cell cycle‐related proteins in SHCBP1 knockdown cells. (B) The intersection of cell cycle pathway‐enriched genes with SHCBP1 Co‐IP followed by MS in PC3M‐IE8 and DU145 cells. (C) SHCBP1 co‐IP and MS revealed a PLK1 representative peptide. (D) Co‐IP analysis indicates the SHCBP1 and PLK1 interaction in G2/M border nocodazole‐synchronized (200 ng/µL) PC‐3 M‐IE8 and DU145 cells. (E) mRNA level of PLK1 in SHCBP1 knockdown cells. (F) Western blot of PLK1 protein and phosphorylation levels in SHCBP1 knockdown cells. (G) Western blot of PLK1‐CDC25C axis phosphorylation in SHCBP1 knockdown cells. (H) Effects of SHCBP1 on proliferation with amplification of wild‐type PLK1 or mutant PLK1 T210D by CCK‐8 assay. (I) Colony formation assay in SHCBP1 knockdown cells treated as indicated in (H). (J, K) Representative images (J) and histogram (K) of cell cycle analysis in SHCBP1 knockdown cells treated as indicated in (H). (L) Western blot of PLK1‐CDC25C axis phosphorylation in SHCBP1 knockdown cells treated as indicated in (H). Values are expressed as mean ± SD, *n* = 3. *p < 0.05; **p < 0.01, ***p < 0.001, ****p < 0.0001.

We found that SHCBP1 regulates G2/M. In order to clarify its regulatory mechanism, we detected the changes in key cell cycle protein of G2/M, and we found decreased levels of Cyclin B1 protein in SHCBP1 knockdown PCa cells (Figure 5A), suggesting that SHCBP1 may regulate G2/M cycle transition by regulating Cyclin B1. Nocodazole synchronization of PC3M‐IE8 and DU145 cells followed by SHCBP1 coimmunoprecipitation (Co‐IP) and mass spectrometry (MS) to determine how SHCBP1 regulates the Cyclin B1/CDK1 complex. The intersection of MS of two cell lines revealed 14 potential SHCBP1 binding proteins. Analysis of these 14 proteins with the cell cycle pathway in Reactome (R‐HSA‐1640170, containing a total of 670 cell cycle enriched gene lists) demonstrated that PLK1 is associated with the cell cycle (Figure 5B,C; Figure S3A,B). The SHCBP1–PLK1 interaction was confirmed by Co‐IP followed by western blotting (Figure 5D). To investigate their molecular function, we measured the mRNA and protein levels of PLK1 in SHCBP1 knockdown cells. SHCBP1 knockdown did not affect PLK1 mRNA or protein levels (Figure 5E,F). Since PLK1 phosphorylation regulates Cyclin B1/CDK1 complex in cell cycle progression, we analyzed PLK1 and its phosphorylation by western blotting and found that the level of PLK1 phosphorylation at threonine 210 (T210) was dramatically reduced (Figure 5F). Therefore, we concluded that SHCBP1 may affect the phosphorylation of PLK1.

SHCBP1 knockdown in PCa decreased the level of PLK1 phosphorylation at T210. Lilia et al.^29^ discovered that PLK1 interacts with the CDC25C phosphatase and promotes its phosphorylation prior to mitotic entry. To explore the downstream consequences of this PLK1‐mediated phosphorylation event, we further conducted a western blot analysis. Our results revealed that the phosphorylation of CDC25C at serine 198 (p‐S198), which is regulated by PLK1, was correspondingly decreased following SHCBP1 knockdown (Figure 5G), so we can infer that SHCBP1 promotes the cell cycle by regulating PLK1 (p‐T210)‐CDC25C (p‐S198) phosphorylation. In SHCBP1 knockdown PCa cells overexpressing PLK1 or PLK1 T210D mutant, we performed CCK‐8 proliferation, plate colony formation, and flow cytometric assays to corroborate our hypothesis. Overexpression of wild‐type PLK1 but not PLK1‐T210D mutant reversed the effects of SHCBP1 knockdown on proliferation and G2/M phase (Figure 5I–K). Moreover, the reduction in PLK1 and CDC25C phosphorylation caused by SHCBP1 knockdown was rescued by overexpression of wild‐type PLK1 instead of the PLK1‐T210D mutant (Figure 5L).

### 2.7 SHCBP1 regulates the nuclear translocation of PLK1

**FIGURE 6:**
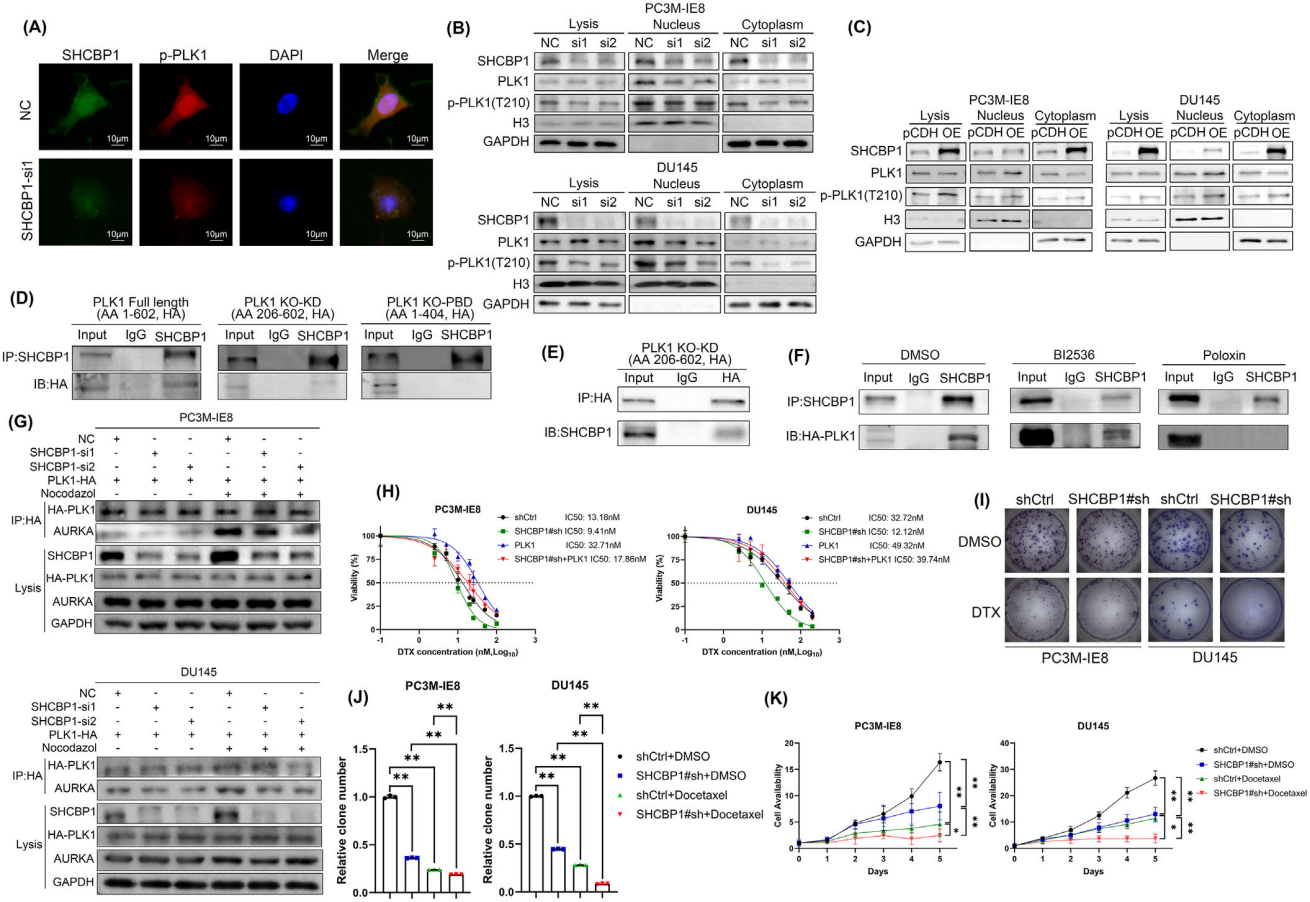
SHCBP1 regulates the interaction of PLK1 and Aurora A through interaction with the PBD of PLK1 and sensitizes PCa cells to docetaxel chemotherapy. (A) Immunofluorescence showed the distribution of SHCBP1 and p‐PLK1 in SHCBP1 knockdown PC3M‐IE8 PCa cells. (B, C) Western blot of plasma and nuclear PLK1 and p‐PLK1 (T210) in SHCBP1‐knockdown or SHCBP1‐overexpressing cells. (D) Co‐IP analysis indicates endogenous SHCBP1 and HA‐tagged PLK1 truncations interaction in nocodazole‐synchronized PC3M‐IE8 cells. (E) Co‐IP analysis indicates HA‐tagged PLK1 truncation KO‐KD interacts with SHCBP1 in nocodazole‐synchronized PC3M‐IE8 cells. (F) Co‐IP analysis indicates endogenous SHCBP1 and PLK1 treated with different PLK1 inhibitors in nocodazole‐synchronized PC3M‐IE8 cells. (G) Co‐IP analysis indicates the PLK1‐AURKA interaction in nocodazole‐synchronized SHCBP1 knockdown PC3M‐IE8 cells. (H) Dose‒response curves of SHCBP1 knockdown PCa cells with or without amplification of PLK1 treated with escalating docetaxel concentrations for 48 h. (I, J) Images and histogram of the colony formation assay in SHCBP1 knockdown PCa cells either with or without PLK1 amplification and treated with 10 nM docetaxel for 48 h. (K) Viability of SHCBP1 knockdown cells treated as indicated in (I, J), *n* = 3/group. *p < 0.05, **p < 0.01.

PLK1 can regulate mitosis by phosphorylating a variety of cell cycle‐associated proteins. Its expression and activation are highly correlated with cell cycle progression. Activated PLK1 can interact with nuclear import proteins such as Importin to induce nuclear translocation.^30^ Since the nuclear translocation of PLK1 is closely related to its phosphorylation/activation, we performed an immunofluorescence (IF) assay of SHCBP1 knockdown cells to examine the nuclear translocation of phosphorylated PLK1. The IF assay revealed a decrease in intracellular p‐PLK1, with the decrease being more pronounced in the nucleus (Figure 6A). In addition, we conducted a nucleoplasmin isolation experiment and found that in SHCBP1 knockdown cells, intracellular p‐PLK1 and nuclear PLK1 levels decreased (Figure 6B). In contrast, after overexpression of SHCBP1, the level of PLK1 phosphorylation was increased, and the expression of PLK1 in the nucleus was increased (Figure 6C).

### 2.8 SHCBP1 regulates the interaction of PLK1 and Aurora A through interaction with the PBD of PLK1

Intramolecular interaction of the kinase domain (KD) and polo‐box domain (PBD) autoinhibits PLK1 during interphase mitosis, but in the early G2 phase, the PBD binds to various upstream substrates, causing a conformational change that recruits Aurora A (AURKA) for phosphorylation modification at T210 of the KD and induces PLK1 kinase activity to phosphorylate downstream substrates. To investigate whether SHCBP1 exerts its cycle‐regulating effect by affecting the regulation of PLK1 phosphorylation by AURKA, we first constructed two domain deletion truncations of PLK1 and subsequently performed Co‐IP assays to examine the interaction region of SHCBP1 and PLK1. The results showed that full‐length HA‐tagged PLK1 and PLK1 with a KD domain deletion bound to SHCBP1, whereas PLK1 with a PBD domain deletion did not (Figure 6D,E). We further treated PC3M‐IE8 PCa cells with two PLK1 inhibitors, BI2536 (an ATP‐competitive inhibitor that dual targets PLK1 and BRD4) and Poloxin (a non‐ATP‐competitive inhibitor that targets the PLK1 PBD structural domain), followed by nocodazole blockade and Co‐IP combined with western blotting. The results showed that HA‐tagged full‐length PLK1 in PLK1 inhibitor BI2536‐treated PCa cells was still able to interact with SHCBP1, while that in PBD inhibitor poloxin‐treated cells were unable to interact with SHCBP1 (Figure 6F). This suggests that the PBD of PLK1 is a potential region by which SHCBP1 interacts with PLK1 and that their interaction was not dependent on the kinase activity of PLK1.

To clarify the mechanism of SHCBP1 phosphorylation and subsequent activation of PLK1, we evaluated the influence of SHCBP1 on the AURKA‐PLK1 interaction. HA‐tagged PLK1 was exogenously expressed in PC3M‐IE8 and DU145 PCa cells. Co‐IP was employed to identify the AURKA‐HA‐tagged PLK1 interaction following nocodazole‐induced G2/M phase arrest. We found that SHCBP1 knockdown did not affect AURKA expression in PCa cells. During the G2/M phase, the interaction between HA‐tagged PLK1 and AURKA was significantly increased, and the interaction was decreased after SHCBP1 knockdown (Figure 6G). Our data demonstrate that SHCBP1 increases AURKA‐mediated PLK1 activation by interacting with the PBD of PLK1.

### 2.9 Knockdown of SHCBP1 improved chemotherapy of PCa cells both in vitro and in vivo

Considering the importance of activated PLK1 for cell mitosis and microtubule synthesis in malignancy, we then investigated whether the knockdown of SHCBP1 could enhance the sensitivity of PC3M‐IE8 and DU145 PCa cells to docetaxel (DTX) chemotherapy. Docetaxel sensitivity in PCa PC3M‐IE8 and DU145 cells was significantly improved by SHCBP1 knockdown, as evidenced by the significantly lower IC50 in the SHCBP1 knockdown group versus the control group. In contrast, docetaxel IC50 was significantly increased by overexpression of PLK1 (Figure 6H). During this time, we treated PCa cells with 10 nM DTX for 48 h and observed a significant decrease in proliferation and clonogenic ability in the SHCBP1 knockdown group, which was especially noticeable in the docetaxel‐treated group (Figure 6I–K). We also verified that SHCBP1 knockdown can increase the chemotherapy sensitivity of docetaxel in vivo through subcutaneous tumor formation experiments in nude mice (Figure S6A–C).

In addition, immunofluorescence staining of microtubules of treated cells demonstrated that SHCBP1 knockdown significantly increased the microtubule toxicity of docetaxel (Figure S4).

## 3 DISCUSSION

We identified differentially expressed genes in PCa bone metastases versus primary tumors using whole‐transcriptome RNA sequencing and bioinformatics. Cell cycle‐related pathways were the main enriched pathways in BM. It is easy to understand that metastasis requires a series of biological changes in tumor cells and that uncontrolled cell proliferation and sustained cell survival contribute to PCa bone metastasis.^31^ PCa bone metastases presented increased SHCBP1 expression, indicating a higher tumor stage and worse prognosis. SHCBP1 was also found to independently predict PCa survival and progression in Cox regression analysis. SHCBP1 may be implicated in PCa bone metastasis. In PCa bone metastasis, SHCBP1 regulates the cell cycle and EMT in vitro.

Cell division is a fundamental way for cells to maintain growth, and it is precisely regulated by various cell cycle‐related proteins. SHCBP1 regulates cell proliferation, differentiation, growth factor signaling, and mitosis.^24^, ^32^ It is involved in MISP‐mediated spindle formation and cytokinesis of gastric cancer cells in the middle and late stages of mitosis.^33^ SHCBP1 expression was considerably higher in quickly dividing cells and lower in dormant or normally differentiated cells.^34^ Similar to many cycle‐related proteins, SHCBP1 is cyclic and peaks in the G2/M phase, confirming that this phase is critical for regulation. Shi et al.^33^ showed that the cell cycle greatly affects SHCBP1 subcellular localization. Before the rupture of the nuclear membrane, mitotic regulators always translocate to the nucleus for cytokinesis.^35^ SHCBP1 knockdown reduces the G2/M phase regulator Cyclin B1, and the transition requires the Cyclin B1/CDK1 complex.^36^ During the G2/M phase, the diphosphatase CDC25C removes the inhibitory phosphorylation sites Thr14 and Thr15 from CDK1, activating it and directing tumor cells into mitosis.^37^ In this study, we found that SHCBP1 interacts with PLK1 and subsequently facilitates the G2/M phase transition.

PLK1 and AURKA are kinases that phosphorylate or dephosphorylate the CDC25C/CDK1/Cyclin B complex to regulate the G2/M phase.^29^, ^38^ PLK1 includes an N‐terminal kinase domain (KD), an intermediate domain, and a C‐terminal PBD. PLK1 conformational alterations can be caused by PBD binding to upstream substrates.^39^ AURKA activates PLK1 by phosphorylating T210 within the T2 loop of the kinase domain,^40^ and a minimal level of Bora/AURKA complex activity is adequate to provide cells with robust PLK1‐facilitated mitosis.^41^ PLK1 and Aurora A kinase coinhibition suppress mitosis in cancer cells.^42^

SHCBP1 knockdown decreased T210 phosphorylation but did not change PLK1 mRNA or protein levels. The phosphorylation of PLK1 can affect its activity and localization, as well as the activity and localization of its target proteins. The phosphorylation of PLK1 can promote the degradation of Wee1 and thus enhance the activity of CDK1.^43^ AURKA and Bora synergistically phosphorylate and activate PLK1, which binds to the phosphatase CDC25C and triggers downstream CDK1 expression.^29^ We found that SHCBP1 knockdown decreased PLK1‐T210 and CDC25C‐SER198 phosphorylation. Moreover, we altered T210 of PLK1 to D210 (T210D) and found that overexpression of PLK1 could restore the SHCBP1 knockdown‐induced decrease in cell proliferation, clonogenic ability, and G2/M phase arrest, but mutant PLK1 could not. Nuclear translocation of SHCBP1 is associated with downstream signaling pathways.^33^, ^44^ PLK1 nuclear translocation was inhibited by SHCBP1 knockdown. We conclude that SHCBP1 can bind to PLK1 through the PLK1‐CDC25C‐CDK1 pathway and affect its phosphorylation/activation, nuclear translocation, and G2/M phase regulation activity.

In the early G2 phase, PLK1 PBD binds to various upstream substrates, causing a conformational change that recruits AURKA for phosphorylation at T210 of the KD and activates its kinase activity to phosphorylate downstream substrates. During interphase mitosis, the KD and PBD autoinhibit PLK1. Our results demonstrated that SHCBP1 interacts with the PBD of PLK1 and may activate PLK1 through this interaction. Bora, Fry, and AIBp have been identified as activators of PLK1 but none can directly phosphorylate PLK1.^17^, ^45^, ^46^ Therefore, we speculate that AURKA, the major kinase that phosphorylates/activates PLK1, may play an irreplaceable role. We synchronized PCa cells and enriched HA‐tagged PLK1 and AURKA by Co‐IP and found that SHCBP1 knockdown reduced the interaction between PLK1 and AURKA. These findings expand our understanding of the mechanisms by which SHCBP1 stimulates and maintains PLK1 expression. Similarly, Shi et al.^33^ confirmed the regulatory role of SHCBP1 in activating MISP phosphorylation as a downstream molecule of PLK1.

In the SHCBP1 knockdown group, the size and number of lesions, bone destruction, and invasiveness of prostate cancer bone metastases in mice were lower than those in the control group. Therefore, we concluded that SHCBP1 regulates bone metastasis not only by regulating the cell cycle but also by regulating cell migration and invasion. Recent studies have shown that not all bone metastases originate from the primary tumor. A study using whole genome sequencing of metastases at different sites in prostate cancer patients revealed the diversity of cell origin within metastases, with cancer cells from other metastases and cancer cells from the primary tumor appearing in the same metastatic site at the same time.^47^ Another exon sequencing study of primary and metastatic sites in 10 breast cancer patients also found that metastases in late‐relapsed tumors were expressed differently from the primary sites, suggesting that metastases in late‐stage tumors may be of diverse origin.^48^ It means that during the formation of bone metastases of prostate cancer cells, there are repeated cell metastasis and colonization processes. Since the metastatic spread of tumor cells is hastened, their capacity to migrate and invade is greatly enhanced by the EMT process. We therefore explored the effect of SHCBP1 on the EMT process of prostate cancer cells. EMT/MET switching is associated with cell cycle control and may affect the balance between cell proliferation and migration.^49^ EMT transcription factors such as SNAIL and ZEB2 can inhibit cell cycle transition by inhibiting the transcription of CCND2 and CCND1, respectively,^50^, ^51^ ZEB1 protein degradation induced by CDK4/6 inhibition blocks breast cancer metastasis.^6^ Although the “Go‐or‐Grow” concept assumes a binary relationship between cell proliferation and migration, this regulatory circuit fine‐tuned the balance of “Go” and “Grow” type cell behavior and produced proliferating cells with an intermediate epithelial–stromal phenotype. These intermediate‐state cells may have a higher metastasis potential. Recently, it has been demonstrated that EMT is not a binary process, but occurs through distinct cellular states.^52^ Cells may move along the EMT–MET continuum, so EMT and MET may not be the fate to split into two conditions, as evidenced by partial EMT.^53^ To our knowledge, after tumor cells have migrated to a new site, such as bone, they must resume proliferation to establish a metastatic niche. Therefore, we conclude that repeated EMT–MET transformation plays an important role in the formation of bone metastases. In sum, our findings suggest that SHCBP1 may have a dual role: initially promoting EMT and migration, and subsequently facilitating the re‐entry of cells into the cell cycle once they have reached their destination. The formation of bone metastases also contributes to the metastasis and implantation of other bone metastases, this could involve the regulation of different sets of target genes at different stages of metastasis. However, it remains puzzling how such SHCBP1 signaling is integrated and requires further research.

SHCBP1 regulates synovial sarcoma and PCa cell motility and invasion in other tumors, according to previous studies.^54^ PLK1 is crucial to tumor cell metastasis and EMT.^55^ Kim et al.^56^ suggested that activated PLK1 phosphorylates β‐catenin at Ser311 and regulates EMT to promote non‐small‐cell lung cancer metastasis. Shin et al.^57^ observed that phosphorylated/activated PLK1 upregulates TGF‐β signaling and increases cell motility and invasiveness through EMT remodeling. In this investigation, we found that SHCBP1 affects tumor cell EMT; hence, its modulation of PLK1 activation may also affect EMT.

Chemotherapy is still used to treat CRPC, notably mCRPC and mCRPC with bone metastasis, but patients have many side effects and low tolerance. At present, taxanes have become the main chemotherapy regimens for CRPC,^58^, ^59^ and only docetaxel and cabazitaxel improve mCRPC survival. Docetaxel and androgen deprivation therapy should be the recommended therapy for males with initial metastases.^60^ Docetaxel is better at relieving bone pain than cabazitaxel. However, almost all mCRPC patients with bone metastases eventually develop docetaxel chemoresistance, and the prognosis of bone metastasis patients remains dismal. The combination of a PLK1 inhibitor and the androgen receptor inhibitor bicalutamide has been found to have a synergistic effect in docetaxel‐resistant PCa cells.^61^ Our studies found that the phosphorylation level of PLK1‐T210 increases when PLK1 is overexpressed (Figure 5L), and the knockdown of SHCBP1 increased the sensitivity of PC3M‐IE8 and DU145 PCa cells to docetaxel. Based on these findings, we speculate that the sensitivity of docetaxel of PCa cells may be mediated by SHCBP1‐induced activation of PLK1. Targeted inhibition of the SHCBP1‐PLK1 axis can not only reduce bone metastasis but also sensitize docetaxel chemotherapy, reducing the increased side effects of multi‐drug combination treatment in PCa patients with bone metastasis.

## 4 CONCLUSION

In conclusion, cell cycle‐related genes are substantially enriched in bone metastasis, and SHCBP1 is overexpressed in PCa tissue and bone metastases. High SHCBP1 expression indicated a higher tumor stage and worse survival. SHCBP1 regulates PCa cell proliferation, metastasis, and G2/M phase transition by affecting PLK1 phosphorylation. Targeted inhibition of SHCBP1‐PLK1 may enhance the chemotherapeutic efficacy of docetaxel in PCa cells. SHCBP1 has prospective clinical applications in the treatment of PCa.

## 5 MATERIALS AND METHODS

### 5.1 Ethics statement

All experiments involving animals were conducted according to the ethical policies and procedures approved by the ethics committee of Sun Yat‐sen University's Institutional Animal Care and Use Committee (approval no. SYSU‐IACUC‐2021‐000041). All experiments involving clinical samples were conducted according to the ethical policies and procedures approved by the ethics committee of Sun Yat‐sen University Ethics Committee (approval no. SYSEC‐KY‐KS‐2020‐201).

### 5.2 Patients and clinical samples

Our study included 104 formalin‐fixed paraffin‐embedded specimens from PCa patients (cohort 1, in situ), 20 specimens from BPH patients, and 27 bone metastasis specimens from PCa patients (Cohort 2, BM) obtained from Sun Yat‐sen University Cancer Center (SYSUCC) between January 2005 and 2014. Two prominent pathologists stained these samples immunohistochemically. The Sun Yat‐sen University Ethics Committee granted the research.

### 5.3 RNA sequencing

Our prior study described RNA extraction and the clinicopathologic characteristics of patients of our RNA sequencing.^27^ *p* < 0.05 and log2(fold change) >1 were considered differentially expressed genes (DEGs). All RNA sequencing data were uploaded to the National Genome Data Center (HRA002360).

### 5.4 Cell lines, cell culture, and cell transfection

PC3M‐IE8, DU145, PC3, 22RV1, C4‐2, LnCap, PC3M‐2B4, and RWPE‐1 cells from the American Typical Culture Collection Center (ATCC) and 293T cells from the Beijing Union Medical College (CRCPUMC) were used in the experimental investigation in this study. All cells were maintained in liquid nitrogen. PC3M‐IE8, PC3, 22RV1, C4‐2, LnCap, and PC3M‐2B4 cells were cultured in RPMI 1640, and DU145 and 293T cell lines were cultured in DMEM (Gibco). RWPE‐1 cell lines were cultured in serum‐free and antibiotic‐free Keratinocyte‐SFM (Gibco). Then, 1% streptomycin‐penicillin and 10% FBS (Sigma Aldrich) were added to PRMI 1640 and DMEM media. Cells were cultured. All cell lines were free of mycoplasma at the time of experimental detection and were tested for misidentification and contamination using short tandem repeat (STR) profiling. Youbio provided the SHCBP1, PLK1, and PLK1 mutant T210D amplification plasmids. GenePharma provided the negative control and SHCBP1 siRNAs. The siRNAs and plasmids were transfected into PC3M‐IE8, DU145, and 293T cells using Lipofectamine RNAiMAX and Lipofectamine 3000, respectively (both from ThermoFisher Scientific), according to the manufacturer's recommendations. Cell Line Authentication. To confirm the identity and authenticity of the PC3M‐IE8 and DU145 cell lines used in this study, STR DNA Genotype Analysis was performed. The obtained profiles matched the known profiles for these cell lines.

### 5.5 Construction of stable cell lines

The CDS of SHCBP1 or PLK1 (and PLK1 mutant) was introduced into the pCDH‐puro plasmid, and its shRNA sequences were introduced into the pLKO.1‐Puro plasmid. The lentivirus was created by cotransfecting 293T cells in 10 cm dishes with target plasmid, pMD2G, and pspAX2 plasmids. A 0.45 µm polyether sulfone membrane was used to collect and filter the 293T cell supernatant 72 h after transfection. PC3M‐IE8 luciferase cells in six‐well plates were infected with untargeted control or SHCBP1#sh lentivirus, and the process was repeated three times every 12 h. Stable knockdown cells were screened using puromycin. SHCBP1 knockdown was measured by qRT‐PCR at least 5 days after the initial infection.

### 5.6 Intersection analysis of differentially expressed genes

We previously established SYSU‐RNA sequencing by whole‐transcriptome sequencing of specimens from six bone metastases, six bone metastatic PCas in situ, and seven primary PCas in situ from our hospital. GSE32269 whole human genome expression oligonucleotide microarrays were downloaded from the GEO portal. Diego^15^ proteomic datasets were downloaded via the portal of Clinical Cancer Research. The DEGs were identified based on the thresholds of *p *< 0.05 and |log2(fold change) |>1.

### 5.7 Cell proliferation assays, colony formation assays, and cell cycle analyses

Cell viability was assessed using colony formation and CCK‐8 assays as previously described. OD was measured at 450 nm after incubating the cells with CCK‐8 reagent at 37°C for 2 h. Cell cycles were identified using flow cytometry as previously described.^62^

### 5.8 Immunofluorescence staining and imaging

Triton X‐100 (Beyotime, P0096‐500 mL) was used to permeabilize transfected cells for 10 min after 30 min of paraformaldehyde fixation. Cells were blocked with QuickBlock Immunostaining Sealer (Beyotime, P0260) and primary antibodies overnight at 4°C. After washing with PBS, they were stained with DyLight‐conjugated secondary antibodies for 1 h and then with DAPI (Beyotime, P0131‐25). Imaging was performed with a laser confocal microscope (Zeiss LSM800 with Airyscan) or fluorescence microscope (Olympus IX71).

### 5.9 RNA extraction and real‐time quantitative polymerase chain reaction

RNA extraction and RT‐qPCR were performed as previously described.^27^ The PrimeScript RT‐qPCR kit (Vazyme) was used to reverse transcribe 1 µg of total RNA to calculate relative mRNA expression based on GAPDH. Table S2 lists the primers used in this study.

### 5.10 Co‐IP and western blot assays

Protein was extracted with Pierce IP lysis buffer (Invitrogen, 87787). Whole‐cell lysates were divided into three parts: one for input and two for IgG incubation at 4°C for 12 h. A/G beads (HY‐K0202, MCE) were spun in the tube at 4°C for 2 h, magnetically removed, and then rinsed five times with lysis buffer. Western blot assays were performed as described.^62^ Enhanced chemiluminescence reagent (Proteintech, PK10003) and SmartChemi 910 were used to identify and image the target protein.

### 5.11 Bone metastasis xenograft models

Shanghai SLAC Co. provided the 4‐week‐old male BALB/c nude mice. Nude mice were injected with 2 × 10^6^ puromycin‐screened SHCBP1 knockdown and corresponding control group PC3M‐IE8‐Luc cells via the intracaudal artery to induce PCa bone metastasis.^28^ There were eight mice in each experimental group. Thereafter, all mice were examined weekly using BLI and radiography (Bruker MI). X‐ray exposure was 10 s and 35 keV. BRUKER MI SE 7.2.1 defined the endpoint event as BLI signals in either group >2 × 10^7^. Mice were monitored until the endpoint event when they were sacrificed by anesthetic overdose and cervical dislocation. Malignant lower limb bone tissue was collected in 10% neutral formalin. Tissues were paraffin‐embedded and decalcified. We have also conducted an additional group that includes a group of normal, healthy mice as a negative control, which did not receive any manipulation.

### 5.12 Prostate cancer xenograft tumor model and therapy

Animal groups and tumor cell implantation: 4‐week‐old male BALB/c nude mice were randomly divided into four groups (six mice per group): (A) shCtrl+DMSO group; (B) SHCBP1#sh+DMSO; (C) shCtrl+docetaxel; (D) SHCBP1#sh+docetaxel. 1 × 10^6^ PC3M‐IE8 cells suspended in 200 µL serum‐free PRMI 1640 were injected subcutaneously into the right flank of each nude mice. When the subcutaneous tumor volume was approximately 100 mm^3^, (A) and (B) groups received tail intravenous injections of DMSO every 3 days, (C) and (D) groups received tail intravenous injections of docetaxel every 3 days (5 mg/kg, Docetaxel RP‐56976, MCE), 4–5 times in total. The general condition of the mice was observed regularly, including weight, diet, and activity. The volume of the tumors was measured regularly using a caliper to measure the length and width of the tumor and calculate the volume using the formula: *V* (mm^3^) = width^2^ (mm^2^) × length (mm)/^2^ × 0.5. Mice were sacrificed by anesthetic overdose and cervical dislocation, the tumors were removed, and the tumor volume and weight were calculated.

### 5.13 Immunohistochemistry

Two independent pathologists, blinded to clinicopathologic information or patient outcome, counted positively stained regions in the three typical fields at 400×. As previously described,^27^ the IRS of protein expression in each segment is the product of the staining intensity score and the positively stained area. High expression was defined as SHCBP1 IRS > 150.

### 5.14 Statistical analysis

SPSS 24.0 and GraphPad Prism 9.3 were used to analyze the data. Quantitative data were expressed using the mean and standard deviation. Independent *t*‐tests were used to compare the groups. One‐way ANOVA and Dunnett's comparison were used to compare quantitative data. The chi‐squared test was used to compare numerical data. Kaplan‒Meier curves were used to calculate progression‐free and overall survival. Hazard ratios and 95% confidence intervals were calculated using the Cox hazard model at *p* < 0.05.

## AUTHOR CONTRIBUTIONS

Conceptualization, supervision, and funding acquisition: Hai Huang, Yiming Lai, and Zhenghui Guo. Writing—original draft, formal analysis, and investigation: Chen Tang and Yongming Chen. Methodology, experiment, and writing—review and editing: Shengmeng Peng and Bisheng Cheng. Resources: Shurui Li. Validation: Jie Zhou and Yongxin Wu. Visualization: Haitao Zhong and Lingfeng Li. All authors have read and approved the final manuscript.

## CONFLICT OF INTEREST STATEMENT

The authors declare no conflict of interest.

## ETHICS STATEMENT

All experiments involving animals were conducted according to the ethical policies and procedures approved by the ethics committee of Sun Yat‐sen University's Institutional Animal Care and Use Committee (approval no.: SYSU‐IACUC‐2021‐000041). All experiments involving clinical samples were conducted according to the ethical policies and procedures approved by the ethics committee of Sun Yat‐sen University Ethics Committee (approval no.: SYSEC‐KY‐KS‐2020‐201). Written informed consent was obtained from all participants.

## Supporting information



Supporting Information

## Data Availability

All datasets involved in this study can be viewed in the Gene Expression Omnibus (GSE32269), National Genomics Data Center (https://ngdc.cncb.ac.cn/) (HRA002360), and portal of Clinical Cancer Research (https://aacrjournals.org/clincancerres/article/24/21/5433/281650/The‐Proteome‐of‐Prostate‐Cancer‐Bone‐Metastasis?searchresult=1). Access to the data underlying results of this study can be provided by the corresponding author upon a reasonable request.
