# Supplementary material for: SHCBP1 is a novel regulator of PLK1 phosphorylation and promotes prostate cancer bone metastasis
Source: MedComm (2020). 2025 Feb 13;6(2):e70082. doi: 10.1002/mco2.70082 (PMC11822462; doi:10.1002/mco2.70082)
Supplement: Supplementary file 1 — Supporting Information [file MCO2-6-e70082-s001.docx]

**SHCBP1 is a Novel Regulator of PLK1 Phosphorylation and Promotes Prostate Cancer Bone Metastasis**

Chen Tang^a,b,c#^, Shengmeng Peng^a,b,d#^, Yongming Chen^e#^, Bisheng Cheng^a,b#^, Shurui Li^a^, Jie Zhou^a,b^, Yongxin Wu^a,b^, Lingfeng Li^a^, Haitao Zhong^a^, Zhenghui Guo^a,b,d*^, Yiming Lai^a,b,d,f*^, Hai Huang^a,b,d*^

^a^Department of Urology, Sun Yat-sen Memorial Hospital, Sun Yat-sen University, Guangzhou, Guangdong, P. R. China

^b^Guangdong Provincial Key Laboratory of Malignant Tumor Epigenetics and Gene Regulation, Sun Yat-sen Memorial Hospital, Sun Yat-sen University, Guangzhou, Guangdong, P. R. China

^c^Department of Urology, Shenzhen Nanshan People's Hospital, Guangdong Shenzhen

^d^Guangdong Provincial Clinical Research Center for Urological Diseases

^e^Beijing Hospital，National Center of Gerontology Institute of Geriatric Medicine, Chinese Academy of Medical Sciences & Peking Union Medical College

^f^Department of Urology, the Fifth Affiliated Hospital of Xinjiang Medical University, Urumqi, Xinjiang, P. R. China

^#^These authors contributed equally.

*Corresponding Authors: Hai Huang, Department of Urology, Sun Yat-sen Memorial Hospital, Sun Yat-sen University, Guangzhou, Guangdong, 510120, P. R. China. E-mail: [huangh9@mail.sysu.edu.cn](mailto:huangh9@mail.sysu.edu.cn); Yiming Lai, E-mail: [laiym3@mail.sysu.edu.cn](mailto:laiym3@mail.sysu.edu.cn); Zhenghui Guo, E-mail: tangch56@mail2.sysu.edu.cn.

**Table S1 Clinicopathological information of prostate cancer patients in the TCGA-PRAD database**

| Variables | TCGA | |
| --- | --- | --- |
|  | Number of cases (%) | |
| Age | ﹤60 | 202(40.6%) |
|  | ≥60 | 296(59.7%) |
| Gleason score | ≤3+4 | 191(38.4%) |
|  | ≥4+3 | 307(61.6%) |
| T stage | Tx | 7(1.4%) |
|  | T2 | 187(37.6%) |
|  | T3 | 293(58.8%) |
|  | T4 | 11(2.2%) |
| N stage | Nx | 74(14.9%) |
|  | N0 | 345(69.3%) |
|  | N1 | 79(15.8%) |
| SHCBP1 | Low | 250(50.2%) |
|  | High | 248(49.8%) |

**Table S2 Basic clinicopathological information of prostate cancer patients with Cohort 1 and Cohort 2**

| Variables | Cohort 1 | | Cohort 2 | |
| --- | --- | --- | --- | --- |
|  | Number of cases (%) | | Number of cases (%) | |
| Age | ﹤60 | 23(22.1%) | ﹤60 | 6(22.2%) |
|  | ≥60 | 81(77.9%) | ≥60 | 21(77.7%) |
| Gleason score | ≤3+4 | 54(51.9%) | ≤3+4 | - |
|  | ≥4+3 | 50(48.1%) | ≥4+3 | - |
| T stage | T2 | 59(56.7%) | T2 | - |
|  | T3 | 29(27.9%) | T3 | - |
|  | T4 | 16(15.4%) | T4 | - |
| N stage | N0 | 91(87.5%) | N0 | - |
|  | N1 | 13(12.5%) | N1 | - |
| M stage | M0 | 98(94.2%) | M0 | 0(0%) |
|  | M1 | 6(5.8%) | M1 | 27(100%) |
| SHCBP1 | Low | 52(50.0%) | Low | 13(48.1%) |
|  | High | 52(50.0%) | High | 14(51.9%) |

**Table S3 Univariate and multivariate cox regression analysis of progression-free survival in the TCGA-PRAD database**

| Variables | Univariate | | Multivariate | |
| --- | --- | --- | --- | --- |
|  | HR (95%CI) | *p-value* | HR (95%CI) | *p-value* |
| **Age (y)** ≥60/﹤60 | 1.43(0.91-2.25) | 0.119 |  |  |
| **Gleason** ≥7(4+3)/≤7(3+4) | 4.95(2.48-9.9) | <0.001** | 3.08(1.48-6.41) | 0.003** |
| **T stage** T3-T4/T2 | 3.98(2.1-7.51) | <0.001** | 2.38(1.21-4.68) | 0.012* |
| **N stage** N1/N0 | 1.92(1.19-3.11) | 0.008** | 1.1(0.67-1.82) | 0.698 |
| **SHCBP1** High/low | 2.35(1.48-3.74) | <0.001** | 1.57(0.97-2.54) | 0.006** |

**Table S4 Univariate and multivariate Cox regression analyses of overall survival (OS) of prostate cancer patients in Cohort1**

| Variables | Univariate | | Multivariate | |
| --- | --- | --- | --- | --- |
|  | HR(95%CI) | *p-value* | HR(95%CI) | *p-value* |
| **Age (y)** ≥60/﹤60 | 1.18(0.46-2.99) | 0.731 |  |  |
| **T stage** T3-T4/T2 | 2.17(0.94-4.99) | 0.069 |  |  |
| **N stage** N1/N0 | 4.88(2.06-11.56) | <0.001** | 3.21(1.25-8.23) | 0.015* |
| **Gleason** ≥7(4+3)/≤7(3+4) | 3.52(1.4-8.83) | 0.007** | 1.61(0.55-4.74) | 0.389 |
| **SHCBP1** High/low | 4.58(1.8-11.71) | 0.001** | 2.8(0.97-8.12) | 0.058 |

Abbreviations: HR = hazard ratio; 95% CI = 95% confidence interval; T stage = tumor stage; N stage= lymph node stage; M stage=metastatic stage. * *p*﹤0.05, ** *p*﹤0.01.

**Table S5 Univariate and multivariate Cox regression analyses of progression-free survival (PFS) for prostate cancer patients in Cohort1**

| Variables | Univariate | | Multivariate | |
| --- | --- | --- | --- | --- |
|  | HR(95%CI) | *p-value* | HR(95%CI) | *p-value* |
| **Age (y)** ≥60/﹤60 | 0.92(0.38-2.19) | 0.846 |  |  |
| **T stage** T3-T4/T2 | 1.78(0.81-3.91) | 0.149 |  |  |
| **N stage** N1/N0 | 4(1.72-9.31) | 0.001** | 2.22(0.89-5.53) | 0.086 |
| **Gleason** ≥7(4+3)/≤7(3+4) | 3.3(1.39-7.81) | 0.007** | 1.61(0.59-4.4) | 0.35 |
| **SHCBP1** High/low | 5.18(2.05-13.08) | 0.001** | 3.29(1.17-9.3) | 0.025* |

Abbreviations: HR = hazard ratio; 95% CI = 95% confidence interval; T stage = tumor stage; N stage= lymph node stage; M stage=metastatic stage. * *p*﹤0.05, ** *p*﹤0.01.

**Table S6 Antibodies used in this study**

| Antibody | Product Code | Manufacturer |
| --- | --- | --- |
| SHCBP1 Antibody | 12672-1-AP | Protein |
| P21 Antibody | A19094 | ABclonal |
| P27 Antibody | YP0201 | Immunoway |
| CCNB1 Antibody | YT1169 | Immunoway |
| HA-tag Antibody (Mouse) | 66006-2-Ig | Proteintech |
| HA-tag Antibody (Rabbit) | YG0003 | Immunoway |
| DYKDDDDK-tag Antibody | 66008-4-Ig | Proteintech |
| PLK1 Antibody | 10305-1-AP | Proteintech |
| CDC25C Antibody | YT0801 | Immunoway |
| CDK1 Antibody | YT0789 | Immunoway |
| E-Cadherin Antibody | 3195S | Cell Signaling Technology |
| N-Cadherin Antibody | YT2988 | Immunoway |
| Vimentin Antibody | 3195S | Servicebio |
| p-PLK1(Thr210) Antibody | YP0964 | Immunoway |
| p-PLK1(S137) Antibody | KA1720C | Immunoway |
| p-CDC25C(S198) Antibody | YP1085 | Immunoway |
| Ki-67 Antibody | GB111499 | Servicebio |
| α-tubulin Antibody | YM3035 | Immunoway |
| H3 Antibody | YT2163 | Immunoway |
| GAPDH Antibody | YN5585 | Immunoway |
| HRP* Goat Anti Mouse IgG(H+L) | RS0001 | Immunoway |
| HRP* Goat Anti Rabbit IgG(H+L) | RS0002 | Immunoway |
| HRP-conjugated Recombinant Rabbit Anti-Mouse IgG, Kappa Light Chain | SA00001-19 | Proteintech |
| HRP-conjugated IgG Fraction Monoclonal Mouse Anti-Rabbit IgG, Light Chain Specific | SA00001-7L | Proteintech |
| Dylight 488, Goat Anti Rabbit IgG | RS23220 | Immunoway |
| DyLight 488, Goat Anti Mouse IgG | RS23210 | Immunoway |
| Dylight 594, Goat Anti Rabbit IgG | RS23420 | Immunoway |
| Dylight 594, Goat Anti Mouse IgG | RS23410 | Immunoway |

**Table S7 Primers used in this study**

| Primer Name | Sequence 5’-3’ |
| --- | --- |
| SHCBP1 Forward | TCTGAGCAAGTCGAGGGAAAT |
| SHCBP1 Reverse | CCCCACAATCCCAACAAACAT |
| PLK1 Forward | CACCAGCACGTCGTAGGATTC |
| PLK1 Reverse | CCGTAGGTAGTATCGGGCCTC |
| GPADH Forward | AAATCCCATCACCATCTTCCAG |
| GPADH Reverse | TGAGTCCTTCCACGATACCAAA |

| Name | Sequence 5’-3’ |
| --- | --- |
| **siRNA** |  |
| si-NC | UUCUCCGAACGUGUCACGUTT |
| SHCBP1-si1 | CCAACCUCUCCCAACUAUATT |
| SHCBP1-si2 | GCAGCAAGCAGCACUACAATT |
| **shRNA** |  |
| sh-Ctrl | CAACAAGATGAAGAGCACCAA |
| SHCBP1#sh | CCAACCTCTCCCAACTATA |

**Table S8 Sequences of siRNA oligos and shRNAs used in this study.**

**Table S9 SHCBP1 from mass spectrometry of SHCBP1 IP in G2/M boundary synchronized PCa cells.**

| Gene Symbol | PC3M-IE8 | | | | DU145 | | | |
| --- | --- | --- | --- | --- | --- | --- | --- | --- |
|  | Coverage  [%] | Peptides | PSMs | Unique Peptides | Coverage [%] | Peptides | PSMs | Unique Peptides |
| HERC2 | 17 | 60 | 63 | 60 | 16 | 54 | 58 | 54 |
| SHCBP1 | 53 | 25 | 30 | 25 | 66 | 30 | 39 | 30 |
| ACTN4 | 27 | 20 | 22 | 8 | 10 | 7 | 7 | 4 |
| LONP1 | 22 | 17 | 19 | 17 | 15 | 11 | 12 | 11 |
| FXR2 | 33 | 13 | 14 | 11 | 45 | 24 | 28 | 21 |
| ACTA1 | 23 | 7 | 14 | 1 | 27 | 9 | 18 | 1 |
| PLK1 | 31 | 11 | 13 | 9 | 21 | 15 | 17 | 15 |
| PARP1 | 14 | 11 | 11 | 11 | 30 | 30 | 35 | 30 |
| HNRNPU | 16 | 9 | 10 | 9 | 20 | 11 | 13 | 11 |
| VIM | 22 | 10 | 10 | 9 | 34 | 17 | 19 | 15 |
| NEURL4 | 7 | 10 | 10 | 10 | 7 | 9 | 10 | 9 |
| UPF1 | 11 | 9 | 9 | 9 | 21 | 22 | 26 | 22 |
| DHX30 | 10 | 9 | 9 | 9 | 10 | 11 | 11 | 11 |
| MSANTD2 | 13 | 7 | 8 | 7 | 16 | 6 | 7 | 6 |
| MOV10 | 10 | 7 | 7 | 7 | 9 | 8 | 9 | 8 |

**Figure legend**

**
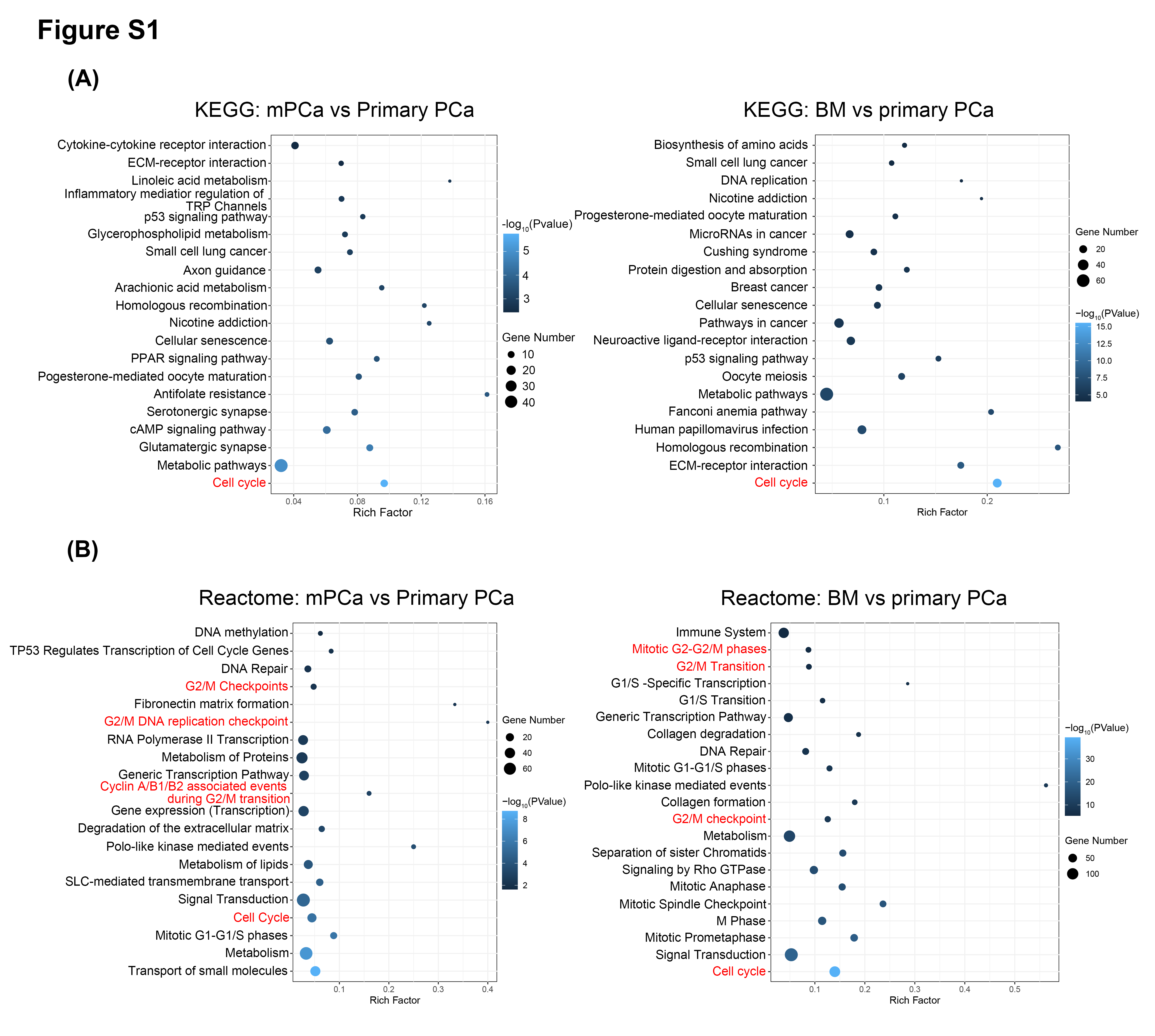
**

**Figure S1 The KEGG and Reactome pathway analysis of upregulated genes in each group. (A)** Bubble chart of KEGG pathway analysis of upregulated genes in mPCa vs Primary PCa and bone metastases vs Primary PCa group. **(B)** Bubble chart of Reactome pathway analysis of upregulated genes in mPCa vs Primary PCa and bone metastases vs Primary PCa group.

**
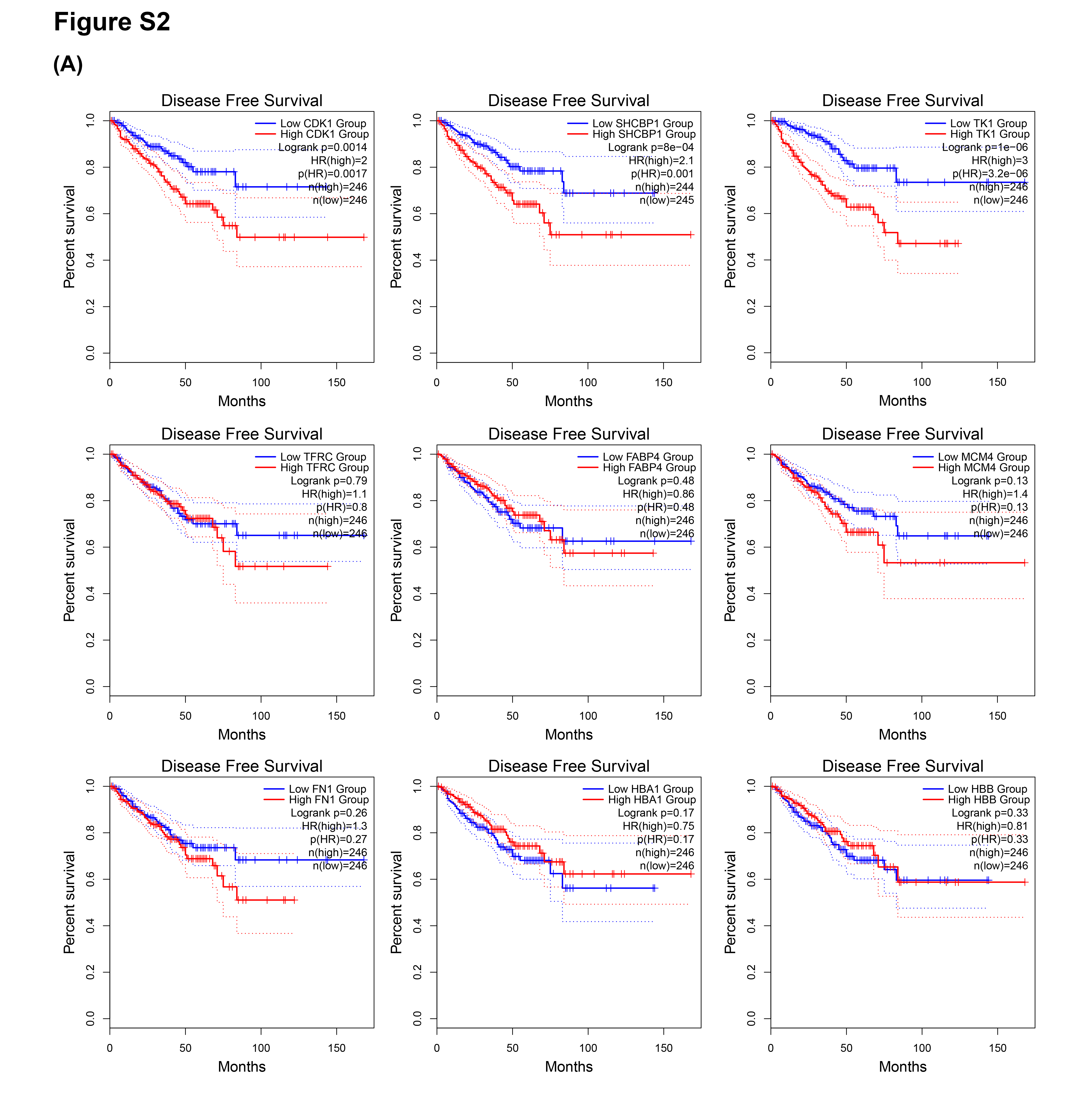
**

**Figure S2 TCGA-PRAD database identified SHCBP1 related to PFS of prostate cancer. (A)** GEPIA2 online tool was used to analyze the relationship between genes and PFS in prostate cancer by intersection analysis in Figure 2A.

**
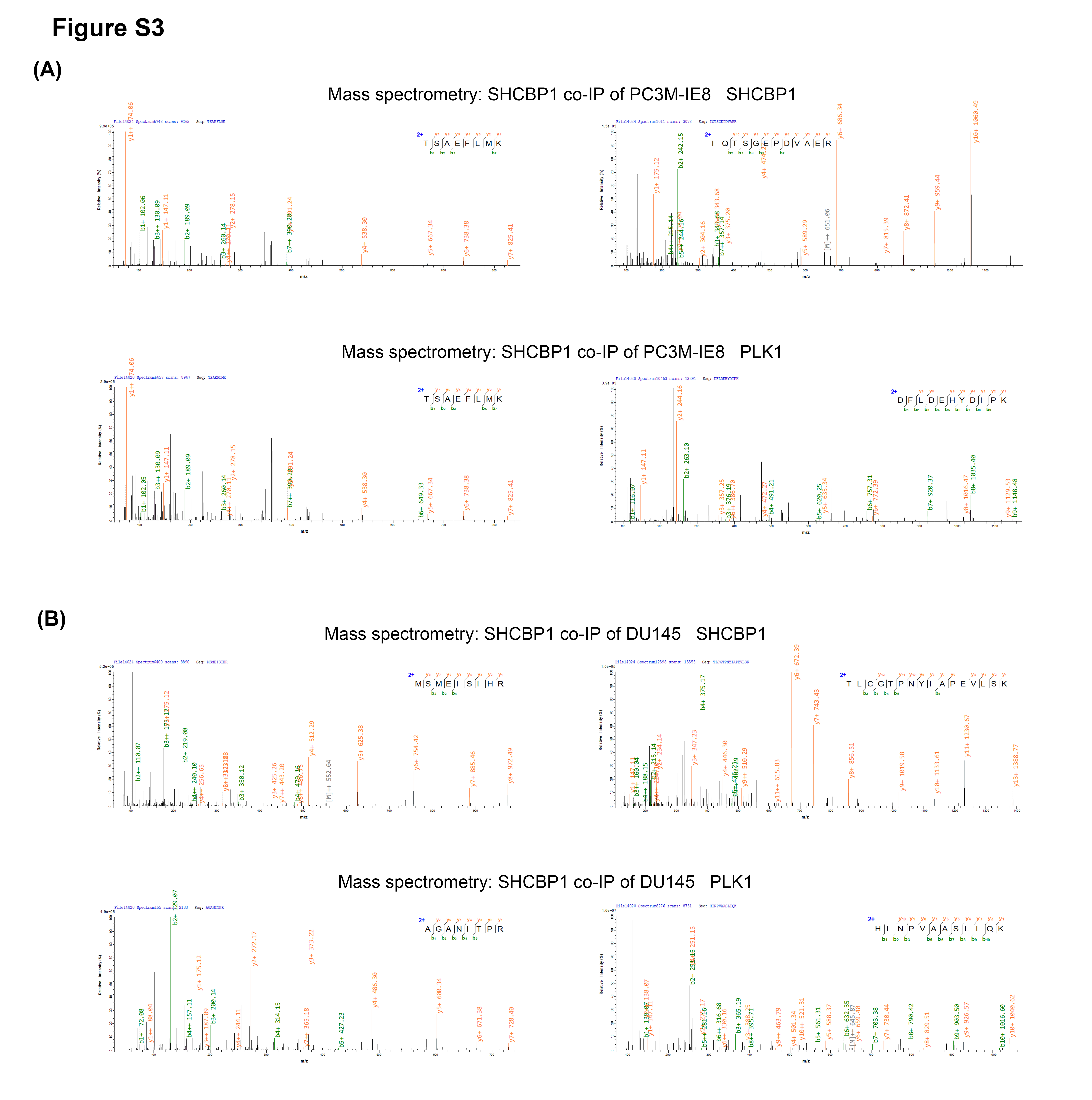
**

**Figure S3 PLK1 is identified to bind with PLK1 in PCa cells. (A)** The representative peptide of SHCBP1 from mass spectrometry of SHCBP1 Co-IP in G2/M boundary synchronized PC3M-IE8 cells. **(B)** The representative peptide of SHCBP1 from mass spectrometry of SHCBP1 Co-IP in G2/M boundary synchronized DU145 cells.

**
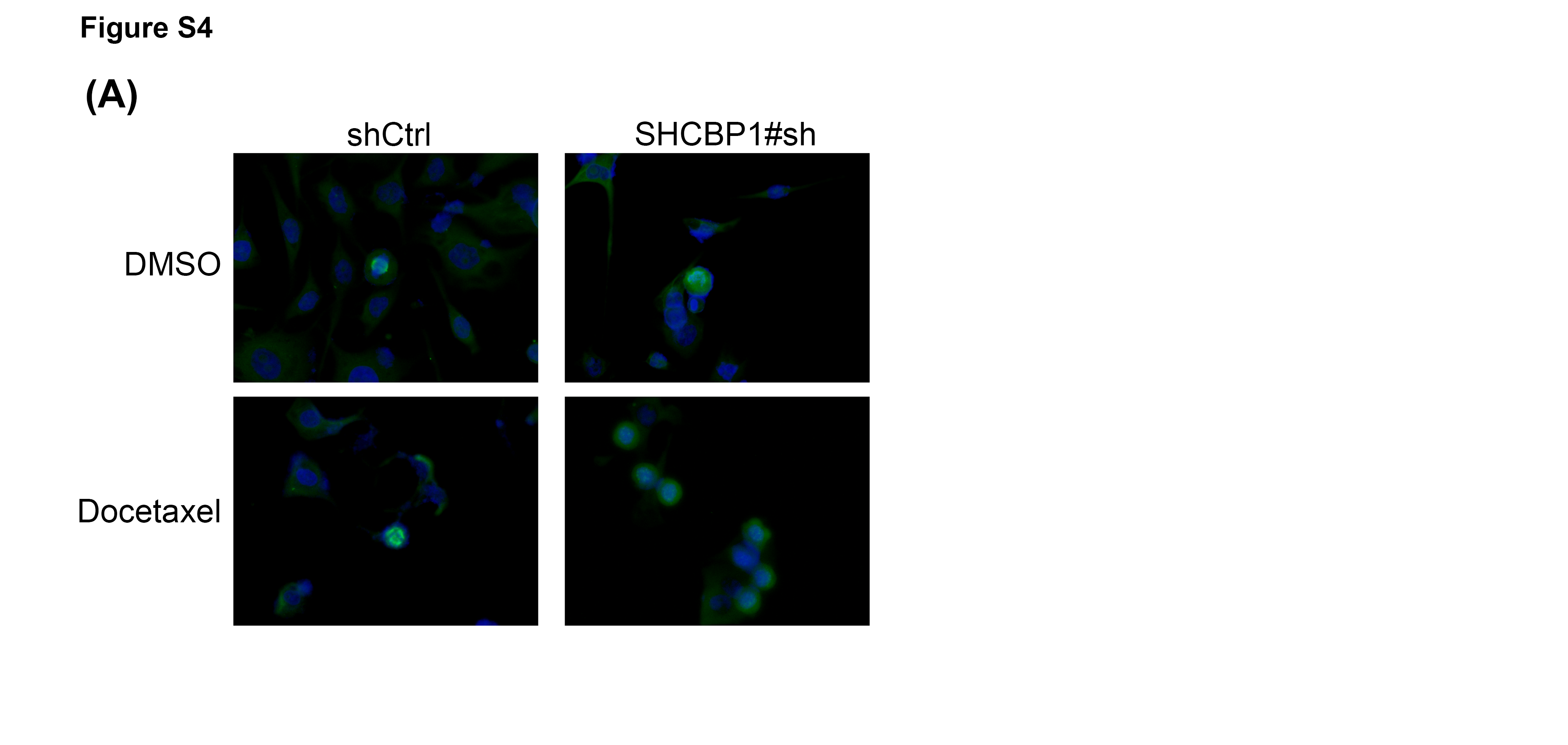
**

**Figure S4** **SHCBP1 knockdown and docetaxel serve synergistically to suppress mitosis in PCa cells. (A)** Immunofluorescence of α-Tubulin (green) and DNA (DAPI, blue) in SHCBP1 knockdown PC3M-IE8 and DU145 cells treated with Docetaxel (10nM) or DMSO for 24h. The white scale bar measures 10 μm.


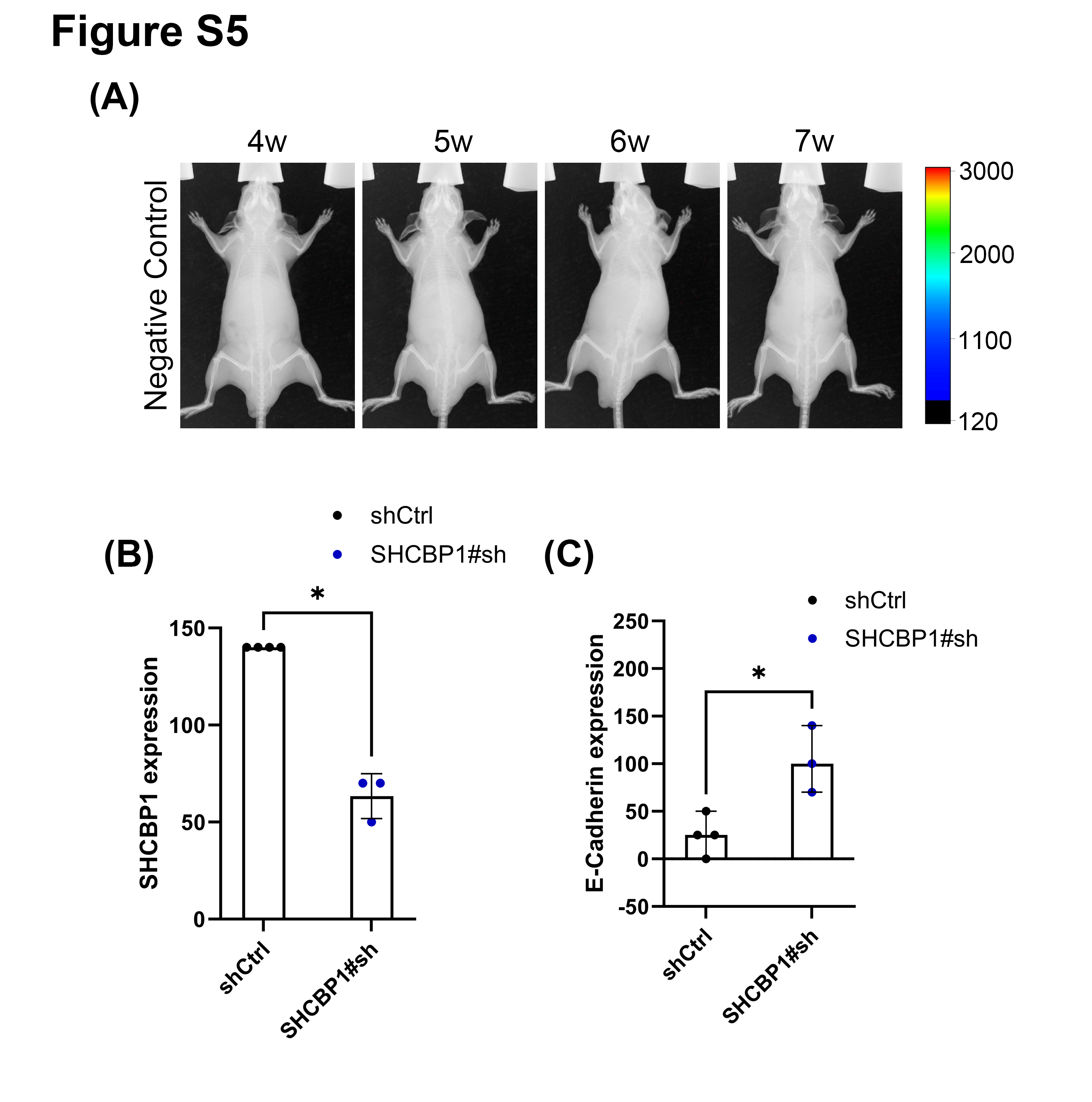


**Figure S5 Bone metastasis xenografts.** **(A)**Normal nude as negative control. **(B)** SHCBP1 expression level in bone metastases. **(C)** E-Cadherin expression level in bone metastases.


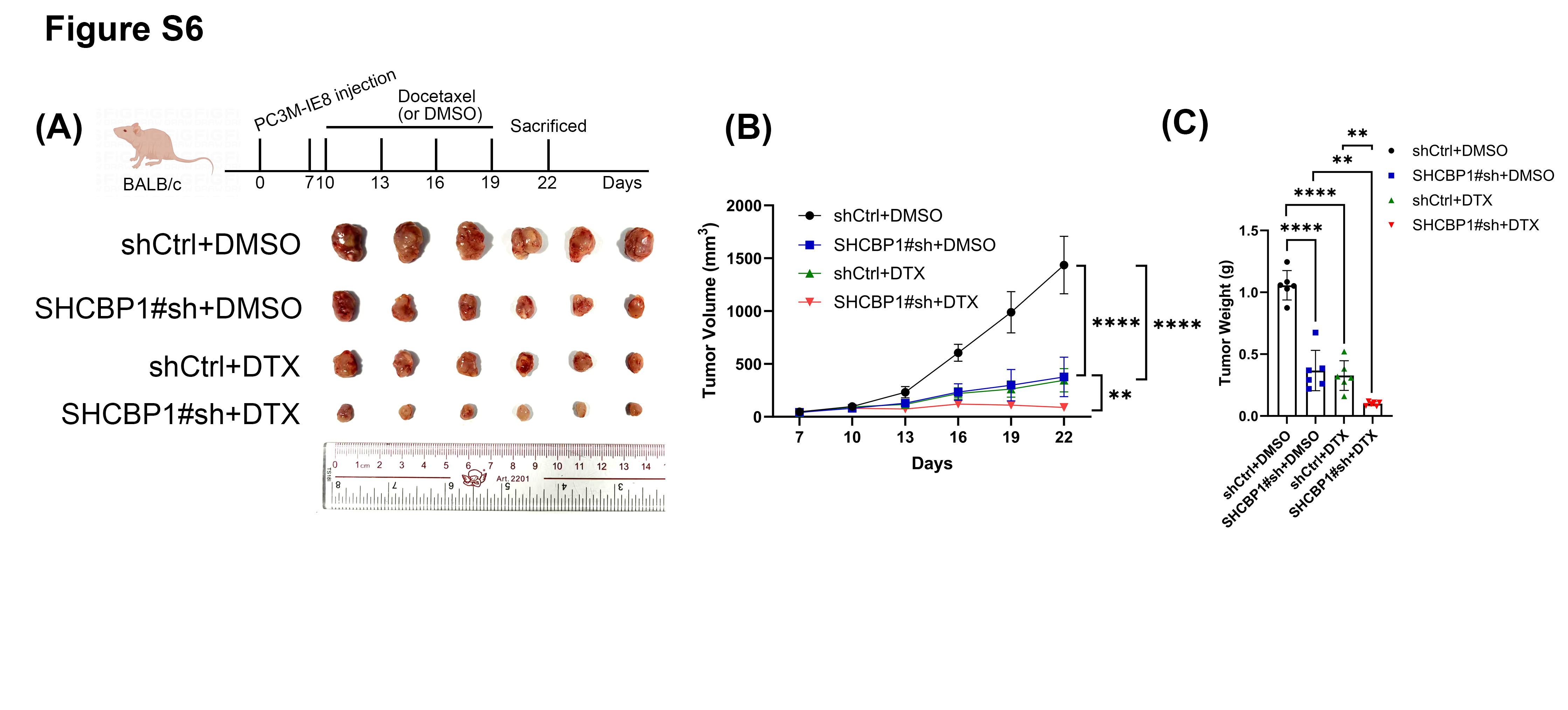


**Figure S6 Knockdown of SHCBP1 improved chemotherapy of PCa cells both in vitro and in vivo.** **(A)**The tumors of the PC3M-IE8 cells subcutaneous xenografts treated with DMSO or Docetaxel in BALB/c nude mice model. **(B)**The tumors volumes were measured regularly every 3 days using the formula: V (mm^3^) = width^2^ (mm^2^) × length (mm)/^2^ ×0.5. **(C)** Comparison of weight of tumors in

the each group, n=6/group.
